# Synthesis and radiolabelled evaluation of novel pyrimidine derivatives as dual α-amylase inhibitors and GIT-targeted molecular imaging probe

**DOI:** 10.1039/d5ra04955e

**Published:** 2025-08-21

**Authors:** Doaa A. Elsayed, Wael Shehta, S. El-Kalyoubi, Adli Selim, Mohammed G. Assy, Omar Metwally, Ahmed A. Al-Kubaisi, Sameer A. Awad, F. Marzook

**Affiliations:** a Department of Chemistry, Faculty of Science, Zagazig University Zagazig 44519 Egypt; b Pharmaceutical Organic Chemistry, Faculty of Pharmacy, Port Said University Port Said 42511 Egypt; c Labelled Compounds Department, Hot Laboratories Center, Egyptian Atomic Energy Authority P.O. Box 13759 Cairo Egypt; d Department of Medical Laboratories Techniques, College of Health and Medical Technology, University of Al Maarif Al Anbar 31001 Iraq doaaatef641995@gmail.com

## Abstract

This study successfully developed synthetic pathways for novel pyrimidine-based compounds for their potential anti-diabetic α-amylase inhibitory activity, aiming at potential antidiabetic applications. Molecular docking techniques showed that compounds 1, 6, and 8 showed the highest molecular docking *in silico* binding affinity, followed by *in vitro* estimation. Compound 1 showed the strongest inhibitory *in vitro* amylase effect, in comparison to the reference drug acarbose. According to *in silico* and *in vitro* combination, a novel radiolabeled compound, ^131^Iodine-Compound 1, was chosen for further labeling study. The radiolabeling factors, including ligand concentration, pH, and reaction time, were adjusted to achieve maximum radiochemical yield and stability. For the *in vivo* behavior, biodistribution studies were estimated in mice up to a 36 hours period. The labelled compound showed significant and prolonged accumulation in the gastrointestinal tract, particularly the stomach and intestine, consistent with its proposed mechanism of enzyme inhibition. A gradual increase in muscle uptake was observed, raising possible insights into side effects reported with similar drugs. These results suggest that compound 1 not only possesses potent amylase-blocking and related effective antidiabetic activity but also holds promise as a molecular probe for molecular dynamic imaging for the GIT system.

## Abstract 2

An ^131^I-labeled α-amylase inhibitor shows exceptional stomach retention (59.6% ID per g) and sustained intestinal uptake, highlighting its promise as a GI-targeted antidiabetic agent and potential GI imaging probe.

## Graphical abstract

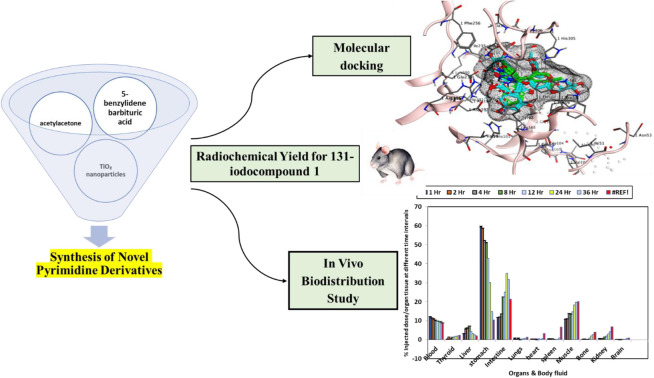

## Introduction

Diabetes mellitus (DM) is recognized as one of the most significant global public health challenges. It is a metabolic disorder characterized by chronic hyperglycemia resulting from defects in insulin secretion, insulin action, or both.^1,2^ There are two main types: Type I diabetes, in which the pancreas produces little or no insulin, and Type II diabetes, where the body becomes resistant to insulin or fails to use it effectively. Although insulin is often still produced in type II cases, its proper utilization is impaired. The variability in insulin response remains an open research question, but contributing factors include obesity, physical inactivity, and genetic predisposition. Type II DM is associated with several comorbidities such as obesity, hypertension, dyslipidemia, and chronic kidney disease, increasing the risk of both acute and chronic complications, including premature mortality.^3^ While lifestyle modifications and weight loss are essential for disease control, maintaining these changes long-term is challenging, and the risk of diabetes may persist even after successful weight management.^4,5^ One therapeutic strategy for DM management involves inhibiting the enzyme α-amylase, which is responsible for catalyzing the breakdown of starch into glucose. Effective α-amylase inhibitors must possess suitable structural features to bind to the enzyme's active site and suppress its catalytic function. Several antidiabetic medications such as metformin, thiazolidinediones (TZDs), sulfonylureas, DPP-4 inhibitors, insulin, and GLP-1 analogs are widely used, yet most are associated with side effects and fail to achieve optimal glycemic control in many patients.^6^ Consequently, there is a growing interest in developing new antidiabetic agents with improved safety and efficacy profiles. Heterocyclic pyrimidine derivatives have shown promise as bioactive scaffolds due to their diverse biological properties.^7–12^ Pyrimidine rings, being fundamental components of nucleic acids, hold particular importance in medicinal chemistry, and several pyrimidine-based compounds have been investigated as potential enzyme inhibitors for diabetes treatment.

Molecular docking and *in silico* modeling can help elucidate the mechanism of action of novel pyrimidine derivatives by predicting their binding interactions with target proteins. Previous studies^13–19^ have employed molecular docking to examine protein-ligand interactions at the atomic level, aiding in the rational design of drug candidates. In this study, synthesized pyrimidine-based compounds were docked against the α-amylase active site (PDB ID: 5E0F)^20^ aiming to identify promising inhibitors.

To further support the biological relevance of the most active compound, a novel radiolabeled derivative (^131^I-pyrimidine) was synthesized. Key radiolabeling parameters, including ligand concentration, pH, reaction temperature, and incubation time, were optimized to ensure high radiochemical yield and *in vitro* stability. *In vitro* α-amylase inhibition assays were conducted to evaluate antidiabetic potential, while *in vivo* biodistribution studies in normal mice were carried out to investigate tissue uptake, pharmacokinetics, and gastrointestinal targeting of the radiolabeled compound.^21–26^ This integrative study combining synthetic chemistry, computational modeling, biological assays, and radiotracing techniques aims to identify and characterize novel pyrimidine-based α-amylase inhibitors with potential dual roles as therapeutic agents and diagnostic imaging probes for diabetes management.

## Experimental

### General information

Starting materials, solvents, and reagents were bought from reputable vendors, including Sigma-Aldrich, Loba, Merck, Acros, and El-Gomhoria. The melting points (°C) were obtained with open capillaries using a Stuart SMP10 melting point instrument and are uncorrected. NMR spectra were acquired on a Bruker high-performance digital FT-NMR spectrometer advance III 400 MHz for ^1^H NMR and 100 MHz for ^13^C NMR (see supplementary information for the spectra). Dimethyl sulfoxide (DMSO)-d_6_ was used as a solvent and tetramethyl silane (TMS) as an internal standard. Radiolabeling: All chemicals and solvents were purchased from Merck Co. Whatman. No. 1 paper chromatography (PC) was exported and supplied by Merck Co. 131-Iodine was purchased from RBF-EAEA. An NaI:Tl scintillator (Scaler Ratemeter SR7 model, England) was used for measuring *γ*-ray radioactivity counts. Double distilled water was used for the preparation of all solutions, and all other chemicals but all reagents consumed in this study were of the analytical grade, used without further purification. α-Amylase Inhibition Assay: The α-amylase inhibition assay was performed using the 3,5-dinitrosalicylic acid (DNSA) method.^21^

### Chemistry

#### 1,1′-(5,8-Dihydroxy-2-oxo-6a-phenyl-2,3,3a1,6a-tetrahydro-1*H*-perimidine-6,7-diyl)bis(ethan-1-one) (1)

A mixture of (0.01 mol) of 5-benzylidene barbituric acid with (0.02 mol) of acetyl acetone and TiO_2_ (1.5 mg) as a nanocatalyst was refluxed in (10 ml) of dimethylformamide as a solvent for 6 hours, then the mixture was filtered to remove TiO_2_, and then the filtrate was cooled down till solid precipitate was formed. Then the mixture was filtrated, the solid precipitate was collected and dried, and then recrystallized from absolute ethanol, and yellow powder was collected. The percentage of the yield is 98%, and the melting point is (230 °C). I.R. (KBr, *υ* cm^−1^): 3492 (OH), 3190 (NH), 3059–2847 (CH for aromatic and aliphatic), 1752 (C=O), 1679 (C=N), 1561, 1492 (C=C aromatic). ^1^H NMR (DMSO-d_6_): *δ* = 2.06 (s, 6H, CH_3_), 2.88 (s, 1H, CH), 6.89 (s, 2H, 2CH=), 7.11–7.35 (m, 5H, Harom), 8.70–8.77 (s, 2H, 2NH), 11.67–11.78 ppm (s, 2H, 2OH); ^13^C NMR (DMSO-d_6_): *δ* = 34.44, 36.62, 50.16, 109.00, 126.76, 129.83, 129.94, 131.08, 143.11, 148.82, 148.92, 162.36, 172.36 ppm. MS (ESI^+^), *m*/*z*: 378.33 (M^+^).

#### Diethyl 2,5,8-trioxo-6a-phenyl-2,3,4,5,6,6a,7,8-octahydro-1*H*-perimidine-6,7-dicarboxylate (2)

A solution of target (A) (.01 mol) and ethyl acetoacetate (.02 mol) and (1.5 mg) of TiO_2_ was refluxed in (10 ml) of DMF for 6 h, then the mixture was filtrated to remove TiO_2_; then it was poured into ice till precipitated, then the mixture was filtrated and the precipitate was collected and dried. Recrystallization occurred by absolute ethanol, then a brown ppt was collected and dried, m.p. is (240 °C). The percentage of the yield is (97.5%). IR. (KBr, *υ* cm^−1^): 3112 (NH), 3057–2837 (CH for aromatic and aliphatic), 1752 (C=O), 1702 (C=O), 1679 (C=O). ^1^H NMR (DMSO-d_6_): *δ* = 1.05–1.08 (t, 6H, 2CH_3_), 2.45–2.50 (q, 4H, 2CH_2_), 3.76–3.78 (d, 1H, CH), 4.00–4.02 (d, 1H, CH), 4.82 (s, 2H, 2CH), 7.13–7.32 (m, 5H, Harom), 11.46 (s, 2H, 2NH). ^13^C NMR (DMSO-d_6_): *δ* = 8.63, 18.56, 48.87, 58.84, 59.24, 100.04, 127.80, 128.11, 128.78, 137.57, 148.79, 162.32, 170.87, 172.77, 207.19, 208.99 ppm.

#### 8-Hydroxy-2,5-dioxo-6a-phenyl-2,3,4,5,6,6a-hexahydro-1*H*-pyrimido[4,5,6-*ij*][2,7]naphthyridine-6,7-dicarbonitrile (3)

A mixture of (0.01 mol) of 5-benzylidene barbituric acid with (0.02 mol) of cyanoacetamide and TiO_2_ (1.5 mg) as a nanocatalyst was refluxed in (10 ml) of dimethylformamide as a solvent for 6 hours, then the mixture was filtered to remove TiO_2_, and then the filtrate was poured onto ice till cooled down, and then solid precipitate was formed. The mixture was filtrated, the solid precipitate was collected and dried, and then recrystallized from absolute ethanol, and yellow powder was collected. The percentage of the yield is (98.6%) and the melting point is (300 °C). IR. (KBr, *υ* cm^−1^): 3473 (OH), 3.317–3219 (3NH), 3054–2929 (CH for aromatic and aliphatic), 2200 (CN), 1623 (C=O). ^1^H NMR (DMSO-d_6_): *δ* = 3.95 (s, 1H, CH), 7.14–7.42 (m, 5H, Harom), 9.14 (s, 2H, 2NH), 10.85 (s, H, NH), 13.34 (s, H, OH).

#### 5,8-Dihydroxy-2-oxo-6a-phenyl-2,3,4,6a-tetrahydro-1*H*-pyrimido[4,5,6-*ij*][2,7]naphthyridine-6,7-dicarboxamide (4)

A solution of target (A) (.01 mol) and monoamide (.02 mol) and (1.5 mg) of TiO_2_ was refluxed in (10 ml) of DMF for 6 h, then the mixture was filtrated to remove TiO_2_. Then it was poured into ice till precipitated, then the mixture was filtrated, and the precipitate was collected and dried. Recrystallization occurred by absolute ethanol, then a yellow ppt was collected and dried, m.p. is (300 °C), and the percentage of the yield is (96%). IR. (KBr, *υ* cm^−1^): 3473 (OH), 3351 (NH), 3159 (NH_2_), 3007–2818 (CH for aromatic and aliphatic), 1615 (C=O). ^1^H NMR (DMSO-d_6_): *δ* = 7.18 (s, 4H, 2NH_2_), 7.57–7.66 (m, 5H, Harom), 8.04–8.06 (s, 3H, 3NH), 11.53 (s, 2H, 2OH).

#### 5,8-Diamino-2-oxo-6a-phenyl-1,6a-dihydro-2*H*-4,9-dioxa-1,3-diazaphenalene-6,7-dicarbonitrile (5)

A mixture of (0.01 mol) of 5-benzylidene barbituric acid with (0.02 mol) of malononitrile and TiO_2_ (1.5 mg) as a nanocatalyst was refluxed in (10 ml)as a solvent for 6 hours, then the mixture was filtered to remove TiO_2_, and then the filtrate was poured onto ice till cooled down. Then solid precipitate was formed, the mixture was filtrated, the solid precipitate was collected and dried, and then recrystallized from absolute ethanol, and yellow powder was collected. The percentage of the yield is (97.3%) and the melting point is (120 °C) .I.R. (KBr, *υ* cm^−1^): 3383 (NH), 3281–3132 (NH_2_), 3107–2964 (CH for aromatic and aliphatic), 2221 (CN), 1677 (C=O). ^1^H NMR (DMSO-d_6_): *δ* = 6.50 (s, 4H, 2NH_2_), 7.43–7.51 (m, 5H, Harom), 9.98 (s, 1H, NH); ^13^C NMR (DMSO-d_6_): *δ* = 29.00, 36.93, 38.99, 39.36, 129.42, 129.36, 129.93, 132.91, 133.03, 149.95, 154.94, 155.33, 157.92, 164.98 ppm. MS (ESI^+^), *m*/*z*: 346.81 (M^+^).

#### 3,3,11,11-Tetramethyl-13b-phenyl-3,4,10,11,12,13b-hexahydro-1*H*-5,9-dioxa-6,8-diazanaphtho[3,2,1-*de*]anthracene-1,7,13(2*H*,6*H*)-trione (6)

A mixture of (0.01 mol) of 5-benzylidene barbituric acid with (0.02 mol) of dimedone and TiO_2_ (1.5 mg) as a nanocatalyst was refluxed in (10 ml) of dimethylformamide as a solvent for 6 hours, then the mixture was filtered to remove TiO_2_, and then the filtrate was poured onto ice till cooled down. Then solid precipitate was formed, the mixture was filtrated, the solid precipitate was collected and dried, and then recrystallized from absolute ethanol, and yellow powder was collected. The percentage of the yield is 98.9%, and the melting point is (200 °C) .I.R. (KBr, *υ* cm^−1^): 3152 (NH), 1761 (C=O), 1678 (C=O). ^1^H NMR (DMSO-d_6_): *δ* = 0.88 (s, 12H, 4CH_3_), 2.05 (s, 4H, 2CH_2_), 2.23 (s, 4H, 2CH_2_), 7.15–7.22 (m, 5H, Harom), 9.89 (s, 1H, NH); ^13^C NMR (DMSO-d_6_): *δ* = 26.68, 28.77, 31.21, 31.85, 50.08, 82.63, 110.54, 114.41, 126.17, 128.03, 139.33, 144.42, 150.67, 156.26, 162.91, 180.03, 195.05 ppm. MS (ESI^+^), *m*/*z*: 458.61 (M^+^).

#### 11b-phenyl-2,3,9,10,11,11b-hexahydro-1*H*-4,8-dioxa-5,7-diazadicyclopenta[*a*,*j*]phenalen-6(5*H*)-one (7)

A solution of target (A) (0.01 mole) and cyclopentanone (0.02 mole) and (1.5 mg) of TiO_2_ was refluxed in (10 ml) of DMF for 6 h, then the mixture was filtered to remove TiO_2_, then it was poured into ice till precipitated, then the mixture was filtered and the precipitate was collected and dried. Recrystallization occurred by absolute ethanol, then a yellow ppt was collected and dried, m.p. is (150 °C), and the percentage of the yield is (96.8%). I.R. (KBr, *υ* cm^−1^): 3191 (NH), 3022–2957 (CH for aromatic and aliphatic), 1683 (C=O). ^1^H NMR (DMSO-d_6_): *δ* = 2.67–2.75 (t, 4H, 2CH_2_), 2.84–2.95 (t, 4H, 2CH_2_), 3.22–3.50 (m, 4H, 2CH_2_), 7.24–7.52 (m, 5H, Harom), 11.45 (s, 1H, NH); ^13^C NMR (DMSO-d_6_): *δ* = 24.28, 30.52, 34.74, 36.17, 80.12, 114.41, 116.52, 128.32, 129.36, 134.28, 135.71, 137.44, 159.42, 162.61, 179.38 ppm.

#### 3a-Phenyl-3a,7-dihydro-1*H*-1,3,4,6,7,9-hexaazaphenalene-2,5,8(3*H*,4*H*,6*H*)-trione (8)

A solution of target (A) (.01 mole) and urea (.02 mole) and (1.5 mg) of TiO_2_ was refluxed in (10 ml) of DMF for 6 h, then the mixture was filtered to remove TiO_2_, then it was poured into ice till precipitated, then the mixture was filtered and the precipitate was collected and dried. Recrystallization occurred by absolute ethanol, then a yellow ppt was collected and dried. The m.p. is (above 300 °C), and the percentage of the yield is (98.6%). I.R. (KBr, *υ* cm^−1^): 3398 (NH), 3022–2821 (CH for aromatic and aliphatic), 1614 (C=O). ^1^H NMR (DMSO-d_6_): *δ* = 7.96–8.07 (m, 5H, Harom), 9.14 (s, 2H, 2NH), 9.38 (s, 2H, 2NH), 10.60 (s, 1H, NH); ^13^C NMR (DMSO-d_6_): *δ* = 77.44, 89.22, 122.30, 129.41, 129.33, 137.31, 143.73, 150.12, 153.40, 163.51, 169.89 ppm.

#### 2a-Phenyl-2a,2a1,7,7a-tetrahydro-6*H*-1,2,3,4,5,7-hexaazacyclopenta[*cd*]inden-6-one (9)

A mixture of 0.01 mol of 5-benzylidene barbituric acid with 0.02 mol of hydrazine and TiO_2_ (1.5 mg) as a nanocatalyst was refluxed in 10 ml of dimethylformamide as a solvent for 6 hours, then the mixture was filtered to remove TiO_2_, and then the filtrate was poured into ice till cooled down. Then solid precipitate was formed, the mixture was filtrated, the solid precipitate was collected and dried, and then recrystallized from absolute ethanol, and yellow powder was collected. The percentage of the yield is 98.1%, and the melting point is (80 °C). I.R. (KBr, *υ* cm^−1^): 3160 (NH), 1646 (C=O). ^1^H NMR (DMSO-d_6_): *δ* = 7.15 (m, 5H, Harom), 8.71 (s, 1H, NH); ^13^C NMR (DMSO-d_6_): *δ* = 89.15, 102.10, 128.34, 129.01, 131.08, 133.78, 161.45, 166.02 ppm.

### ADMET properties

The tools utilized for POM analysis, particularly MOLINSPIRATION^27^ and ProTox-II,^28^ follow the same fundamental premise. The molecular structure is first established by drawing it or inputting its SMILES code. The specific parameters are then calculated using the fragment system.,^29,30^ followed by molecular modeling for the most active chemicals using the molecular operating environment software.^31–35^

### Preparation of protein

Chemdraw 12.0 was used to sketch the molecular modeling for the most active chemicals to be docked by using Molecular Operating Environment software (2022). The results were refined using the London DG force and force field energy. MMFF 94 (Merck molecular force field 94) was used to complete all minimizations until a root mean square deviation (RMSD) gradient of 0.1 kcal mol^−1^ A^−1^ was reached,^36–38^ and the partial charges were determined automatically. The MOE software's scoring function, dock function (*S*, kcal mol^−1^), was used to determine the ligand's binding affinity. The X-ray crystal structure of the enzyme was retrieved in PDB format from the protein data bank (PDB ID: 5E0F, resolution: 1.40) (https://www.rcsb.org/structure/5E0F). The enzyme was prepared for docking studies by removing water, adding all hydrogen bonds, fixing potential, and generating dummy atoms from the resulting alpha spheres^33,39^ then analyzing the ligand's interaction with the active site's amino acids. The best docking score is obtained as the most negative value for the active ligands.^40,41^

### Radiolabeling procedure

Radiolabeling was performed using the direct labeling method, with the pH of the preparation adjusted, followed by incubation at room temperature for a specified reaction time. The effects of ligand concentrations (1 mg ml^−1^ ethanol), reaction time, and pH on radiolabeling efficiency were optimized to establish optimal reaction conditions.^21–26^

The chemical identity and radiochemical purity of [^131^I]–Compound 1 were confirmed using co-thin layer chromatography (co-TLC) with the corresponding non-radioactive standard. Silica gel TLC plates were developed in chloroform:methanol (3 : 1, v/v) as the mobile phase. [^131^I]–Compound 1 exhibited an *R*_f_ value of 0.70, identical to that of the cold standard, whereas free iodide remained at the origin (*R*_f_ = 0.0). This result confirms the successful labeling of Compound 1 and the absence of unbound [^131^I], in agreement with previously reported TLC-based radiolabeling confirmation methods.^42^

### Animal studies

Quantitative biodistribution studies were carried out using male Swiss albino mice (20–25 g), purchased from the Animal House of the labelled compounds department. Mice were kept under standard laboratory conditions ad libitum. Minimizing animal scarification Efforts were carefully made to minimize the number of animals utilized, under EAEA license number P/99A/24 from NCRRT. Mice were kept in a comfortable environment, including nesting materials and tunnels, with suitable cages to reduce stress.^26,43,44^

Biodistribution studies were conducted *via* intravenous (IV) administration in normal mice. Each mouse received an oral injection of 0.05 ml containing 0.74 MBq per μmol of the radiolabeled complex using yellow tips of an automated micropipette. Mice were maintained for predefined time intervals, after which tissue distribution was assessed. Mice were sacrificed at 1, 2, 4, 8, 12-, 16-, 24-, and 36 hours post-injection (p.i). A total number of 40 animals were divided into 8 groups, with 5 mice per time interval. Organs were dissected and weighed, and their radioactivity was counted by using a *γ*-ray scintillation counter. The percentage of injected dose (% ID per g) was calculated for each sample using a standard equation. At each time point, mice were weighed and anesthetized using a ketamine/xylazine combination before being sacrificed *via* cervical dislocation. The administered anesthesia doses were Ketamine: 75–100 mg kg^−1^ and Xylazine: 5–10 mg kg^−1^. Cervical dislocation was used for scarification to confirm merciful death before tissue collection. All carcasses and tissues containing radioactive material were handled in compliance with the Egyptian Atomic Energy Authority guidelines.^45–48^

### Statistical treatment of biological data

#### Radiolabeling

Optimized at pH 4, 200 μg ml^−1^ Compound 1, 30 min, 25 °C (*n* = 3, *p* < 0.01).

#### α-Amylase assay

IC_50_ calculated *via* nonlinear regression (GraphPad Prism v9.0), triplicate experiments.

#### Biodistribution

Data expressed as mean ± SEM (*n* = 5/group), analyzed by ANOVA + Tukey's test.

## Results and discussion

### Chemistry

**Scheme 1:**
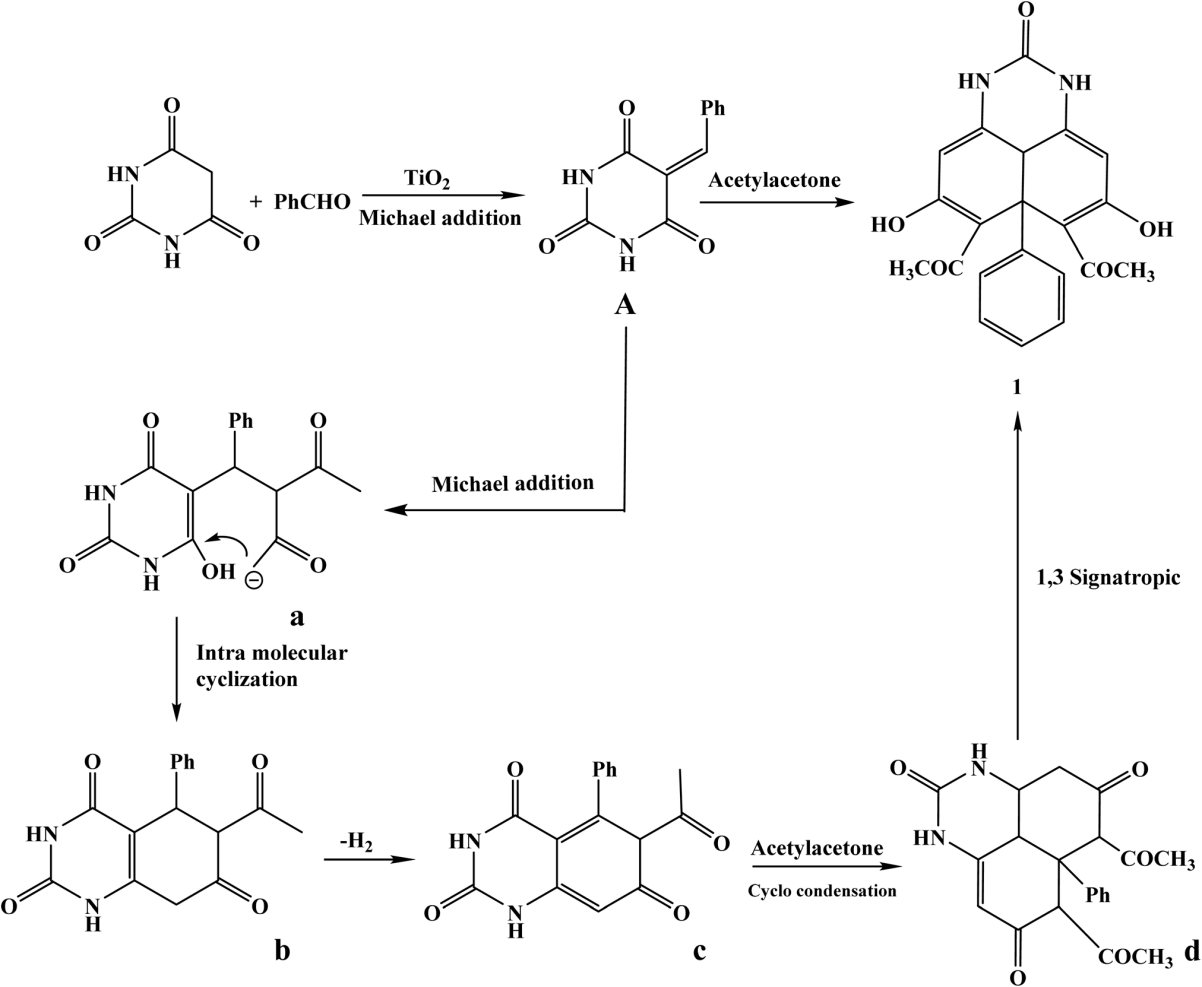
Cyclization of 5-benzyldine barbituric acid with acetylacetone.

1,1′-(5,8-Dihydroxy-2-oxo-6a-phenyl-2,3,3a1,6a-tetrahydro-1*H*-perimidine-6,7-diyl)bis(ethan-1-one), a novel perimidine derivative, was successfully synthesized in this study using a one-pot multicomponent reaction that involved 5-benzylidene barbituric acid, acetylacetone, and TiO_2_ nanoparticles as a nanocatalyst under reflux in dimethylformamide (DMF). The final polyfunctionalized heterocyclic scaffold is the result of a sequential Michael addition, intramolecular cyclization, and 1,3-sigmatropic rearrangement, as shown in the suggested scheme. Because of the nanomaterial's high catalytic effectiveness and increased surface area, which facilitate the activation of carbonyl groups and promote the synthesis of intermediates, the introduction of TiO_2_ nanoparticles greatly raised the reaction yield (98%) and decreased the reaction time, Scheme 1. Several spectroscopic methods were used to validate the synthesized compound's structure. The O–H and N–H stretching vibrations are represented by the distinctive absorption bands in the infrared spectra, which were found at 3492 cm^−1^ and 3190 cm^−1^, respectively. While the peaks at 1561 and 1492 cm^−1^ verify the presence of aromatic C=C bonds, the strong bands at 1752 cm^−1^ and 1679 cm^−1^ are ascribed to the stretching vibrations of the conjugated carbonyl (C=O) and imine (C=N) functionalities. Acetyl methyl and methine groups were detected by the ^1^H NMR spectra (DMSO-d_6_), which showed singlets at *δ* = 2.06 ppm (6H, CH_3_) and *δ* = 2.88 ppm (1H, methine proton). The aromatic protons resonated as a multiplet in the range of *δ* = 7.11–7.35 ppm (5H), whereas the olefinic protons showed up as singlets at *δ* = 6.89 ppm (2H). Downfield signals at *δ* = 8.70–8.77 ppm (2H, NH) and *δ* = 11.67–11.78 ppm (2H, OH) verified that the heterocyclic ring included hydrogen-bonded protons. Additionally, signals corresponding to methyl (*δ* = 34.44, 36.62), methine (*δ* = 50.16), aromatic/olefinic carbons (*δ* = 109.00–143.11), imine, and carbonyl carbons (*δ* = 148.82–172.36) were found in the ^13^C NMR spectra, which confirmed the given structure. The ESI^+^ mass spectrum's molecular ion peak, which is detected at *m*/*z* = 378.33 (M^+^), validates the target compound's effective synthesis by confirming the product's molecular weight.

**Scheme 2:**
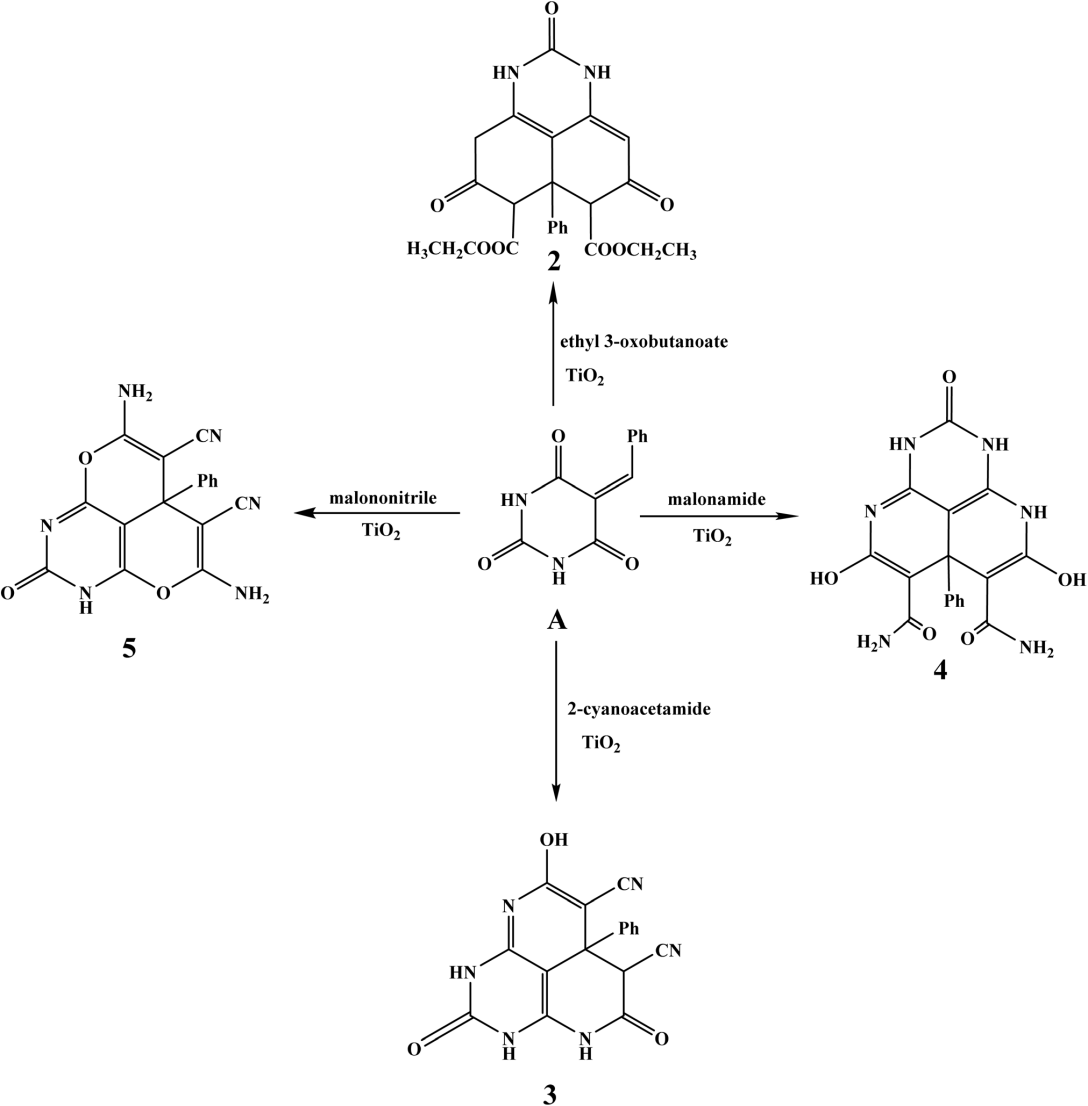
Cyclization of 5-benzylidenepyrimidine-2,4,6(1*H*,3*H*,5*H*)-trione with reagent bearing active methylene group.

Using 5-benzylidene barbituric acid as the primary substrate, a number of pyrimidine derivatives were effectively produced by multicomponent reactions that were TiO_2_ nanocatalyzed. Target compounds (2–5) are produced under moderate circumstances utilizing TiO_2_ nanoparticles (1.5 mg) as an effective and recyclable heterogeneous catalyst in DMF at reflux for 6 hours, as shown in the schematic approach 2. Because of their large surface area, Lewis acidity, and superior dispersion in polar fluids, TiO_2_ nanoparticles were essential in increasing reaction speeds and yields (96–98.6%). Through the processes of Knoevenagel condensation, Michael addition, and intramolecular cyclization, these properties promoted the creation of pyrimido[4,5,6-*ij*][2,7]naphthyridine and perimidine frameworks by effectively activating carbonyl and nitrile groups. The presence of multiple carbonyl functionalities was confirmed by the characteristic IR absorption bands for NH (3112 cm^−1^), aliphatic/aromatic C–H (3057–2837 cm^−1^), and strong carbonyl stretches (1752, 1702, 1679 cm^−1^) displayed by compound 2, diethyl 2,5,8-trioxo-6a-phenyl-2,3,4,5,6,6a,7,8-octahydro-1*H*-perimidine-6,7-dicarboxylate. Ethyl groups were represented as triplets and quartets in the ^1^H NMR spectrum at *δ* 1.05–2.50 ppm, while aromatic protons were seen as a multiplet at *δ* 7.13–7.32 ppm. NH groups were detected in two singlets at *δ* 11.46 ppm. The structure was further validated by ^13^C NMR, which showed distinctive signals for aliphatic, aromatic, and carbonyl (*δ* ∼172–209 ppm) carbons. 8-Hydroxy-2,5-dioxo-6a-phenyl-2,3,4,5,6,6a-hexahydro-1*H*-pyrimido [4,5,6-*ij*] is compound 3. The stretching vibrations of hydroxyl (3473 cm^−1^), NH (3219–3317 cm^−1^), aromatic/aliphatic C–H (3054–2929 cm^−1^), and nitrile (2200 cm^−1^) were clearly visible in [2,7]naphthyridine-6,7-dicarbonitrile. The cyclization and nitrile substitution were verified by the IR and NMR. A tricyclic naphthyridine system was supported by the ^1^H NMR spectra, which revealed a downfield singlet for OH at *δ* 13.34 ppm and extra NH protons at *δ* 9.14 and 10.85 ppm. The effective insertion of amide functionalities was demonstrated by Compound 4, a dihydroxy-dicarboxamide derivative, which showed IR absorptions for OH (3473 cm^−1^), NH and NH_2_ groups (3351, 3159 cm^−1^), and C=O (1615 cm^−1^). In addition to aromatic protons, the ^1^H NMR verified this with singlets at *δ* 7.18 ppm for NH_2_ and *δ* 11.53 ppm for hydroxyl groups. The high yield (96%) and melting point provided additional evidence of the nanocatalyst's effectiveness in creating highly functionalized heterocycles. In addition to a prominent nitrile band at 2221 cm^−1^ and C=O at 1677 cm^−1^, compound 5, the diamino-dicyano derivative, showed IR bands for NH and NH_2_ at 3383 and 3281–3132 cm^−1^. NH at *δ* 9.98 ppm, aromatic multiplet at *δ* 7.43–7.51 ppm, and NH_2_ protons at *δ* 6.50 ppm were all visible in the ^1^H NMR spectrum. Signals for nitrile, carbonyl, and aromatic carbons were detected by ^13^C NMR. The estimated molecular ion peak and the mass spectrum (*m*/*z* = 346.81) agreed, indicating molecular integrity (Scheme 2).

**Scheme 3:**
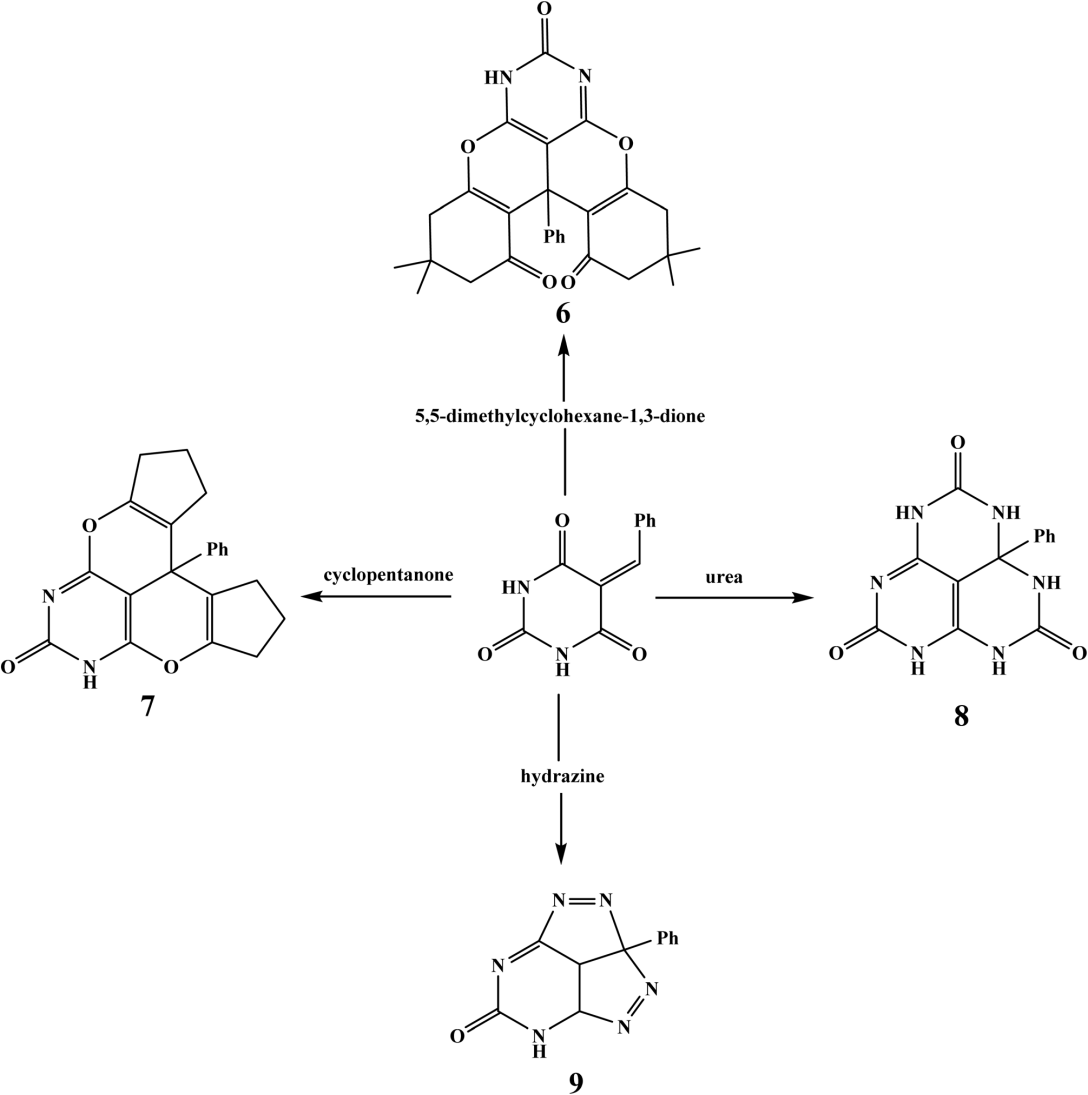
Heterocyclization of 25-benzylidenepyrimidine-2,4,6(1*H*,3*H*,5*H*)-trione with cyclic ketones and binucleophilic nitrogen.

Using 5-benzylidene barbituric acid as a crucial intermediate and a variety of nucleophilic partners, including dimedone, cyclopentanone, urea, and hydrazine hydrate, a multicomponent reaction was used to create a number of new fused heterocyclic systems (compounds 6–9). TiO_2_ nanoparticles (1.5 mg) were used as an effective nanocatalyst. For six hours, reactions were conducted in DMF under reflux conditions. Because of their large surface area and improved catalytic qualities, which allow for efficient interaction with reactants and facilitate bond formation *via* Knoevenagel–Michael-type sequences and intramolecular cyclizations, TiO_2_ NPs greatly increased yield (96.8–98.9%) and decreased reaction time. IR revealed a wide NH band at 3152 cm^−1^ and significant C=O absorptions at 1761 and 1678 cm^−1^ for compound 6, which was created by interaction with dimedone. In addition to a singlet for NH at 9.89 ppm, the ^1^H NMR spectra verified the existence of four methyl groups (*δ* = 0.88 ppm, 12H), methylene protons (*δ* = 2.05, 2.23 ppm), and aromatic protons (*δ* = 7.15–7.22 ppm). A molecular ion peak at *m*/*z* 458.61 was visible in the MS spectra, which was in line with the predicted mass. The IR bands for NH (3191 cm^−1^), aromatic/aliphatic C–H (3022–2957 cm^−1^), and C=O (1683 cm^−1^) were seen in compound 7, which was created using cyclopentanone. The cyclopentyl CH_2_ protons (*δ* = 2.67–3.50 ppm) were found to have complex multiplets in ^1^H NMR, coupled with aromatic protons and a downfield NH signal at 11.45 ppm. The aliphatic CH_2_, quaternary carbons, and carbonyl groups were represented by peaks in the ^13^C NMR spectra, which supported the production of a fused dicyclopenta[*a*,*j*]phenalene skeleton. In the IR spectra, compound 8, which was the result of a reaction with urea, had a high NH absorption at 3398 cm^−1^ and C=O at 1614 cm^−1^. Multiple NH protons in a polyazaphenalene framework were indicated by the distinctive NH signals at *δ* = 9.14, 9.38, and 10.60 ppm in the ^1^H NMR spectra. The appearance of aromatic protons occurred around *δ* = 7.96–8.07 ppm. The hexacyclic trione structure was supported by the ^13^C NMR spectra, which verified the existence of carbonyl carbons, heteroaromatic rings, and sp^2^ carbons. In the infrared spectra, compound 9, which was made using hydrazine, displayed a C=O band at 1646 cm^−1^ and a single NH stretch at 3160 cm^−1^. Aromatic protons at *δ* = 7.15 ppm and a singlet NH at 8.71 ppm were detected by the ^1^H NMR. The development of a hexaazacyclopenta[*cd*]indene framework was confirmed by the carbon spectra, which showed peaks at *δ* = 89.15 and 102.10 ppm that corresponded to freshly produced heterocyclic carbons together with aromatic and carbonyl carbon signals (Scheme 3).

### Analysis of ADME characteristics of ligand

POM analysis and other comparable procedures are useful for identifying various physicochemical properties and anticipating a molecule's biological activity, ADME parameters, and toxicity. Compounds 1, 2, and 6 underwent a modified POM analysis using the MOLINSPIRATION tools.

### Molinsipration calculation

MOLINSPIRATION offers a comprehensive range of computational biology applications to assist with molecular manipulation and processing. These tools include SMILES and Sdfile conversion, molecule normalization, tautomer generation, molecule fragmentation, calculation of various molecular properties required for QSAR, molecular modeling and drug design, high-quality molecule depiction, and molecular database tools that aid in substructure and similarity searches.^15,49^

Table 1 shows the predicted pharmacokinetic/molinspiration characteristics for compounds produced 1, 2, and 6. Using Molinspiration online screening, virtually all of the compounds created have potential biological activity, as evidenced by the docking parameters in Table 2, which highlight drug-like characteristics against kinase inhibitors, proteases, and enzyme inhibitors. The calculated distribution of activity scores (version 2022.08) is contrasted with scores for GPCR ligands, kinase inhibitors, ion channel modulators, nuclear receptor ligands, protease inhibitors, and other enzyme targets. These ratings include scores for more than 100 000 typical drug-like substances. The score allows for effective distinction of active and inactive compounds.

**Table 1:** Physicochemical properties of the synthesized compounds^a^

Compound	Mi Log *P*	TPSA	*n*-atoms	M.W.	*n*ON	*n*OHNH	*n*-violations	*n*-rotb	Volume
1	1.96	115.72	28	378.38	7	4	0	3	325.43
2	2.74	141.72	32	438.44	9	4	0	7	307.00
6	4.49	98.36	34	458.51	7	1	0	1	405.63

a Abbreviations: Mi log *P*, logarithm of partition coefficient of compound between *n*-octanol and water; MV, molecular volume; MW, molecular weight; *n* atoms, number of atoms; *n*-ON acceptors, number of hydrogen bond acceptors; *n*-OHNH donors, number of hydrogen bonds donors; *n* rotb, number ofroutable bonds; *n* violations, number of violations; TPSA, topological polar surface area.

**Table 2:** Physicochemical Molinspiration bioactivity score

Compound	GPCR ligand	Ion channel modulator	Kinase inhibitor	Nuclear receptor ligand	Protease inhibitor	Enzyme inhibitor
1	−0.35	−0.28	−0.53	−0.07	−0.31	−0.23
2	−0.21	−0.25	−0.39	−0.12	−0.30	−0.14
6	−0.31	−0.45	−0.70	−0.31	−0.47	−0.23

### ProTox-II tool

**Fig. 1:**
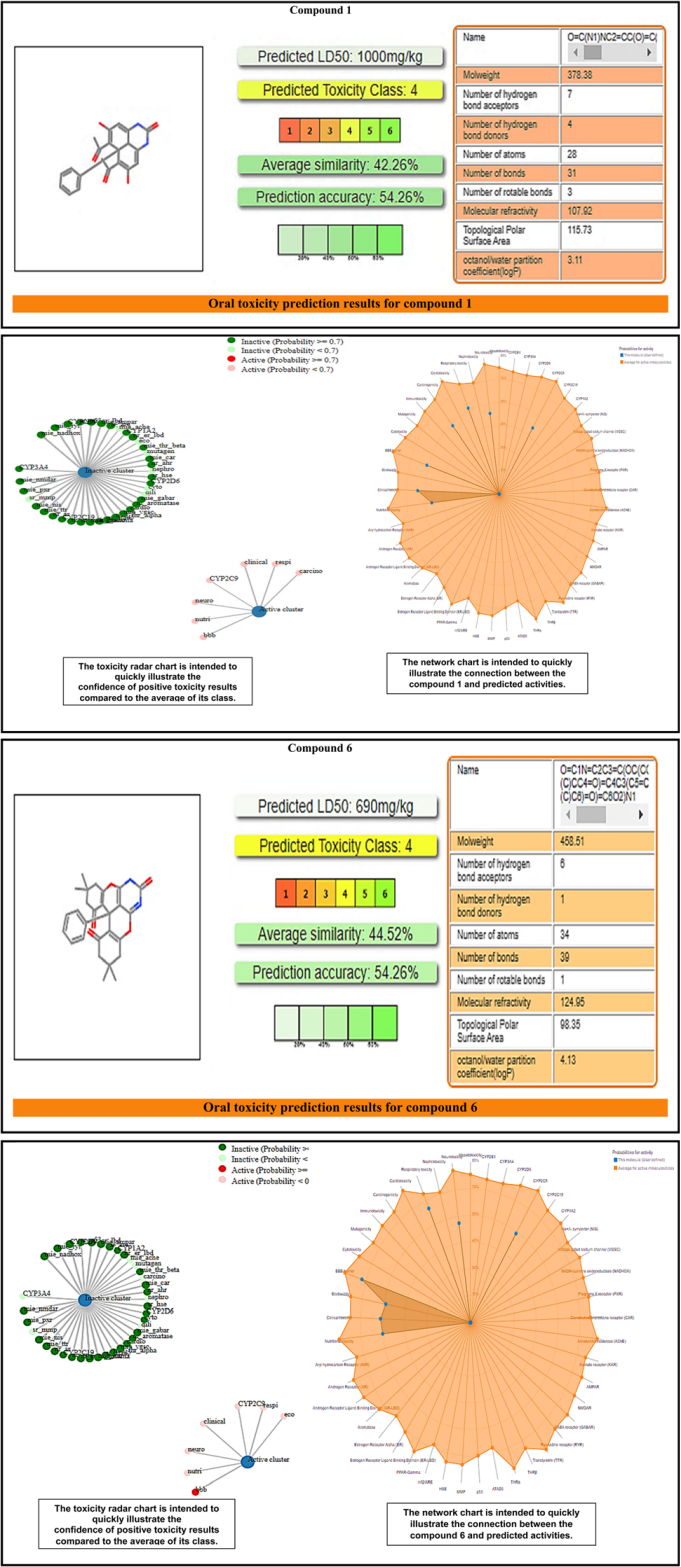
Displays the comparison of chemicals 1 and 6 to those in the dataset on the ProtoxII virtual lab.

ProtoxII virtual lab for the study of small molecule toxicity. Identifying chemical toxicity is an important stage in the development of novel medications. According to ProTox-II, the oral LD_50_ values for the two compounds in a rat model vary from 690 to 1000 mg kg^−1^, with quercetin having the lowest value and (1s,4s)-Eucalyptol having the highest. Fig. 1 shows a comparison of chemicals 1 and 6 to those in the dataset.^15^ Toxicology radar Fig. 1 is meant to immediately demonstrate the confidence of favorable toxicity data when compared to the average of its class for chemicals 1 and 6.

### Molecular docking

**Fig. 2:**
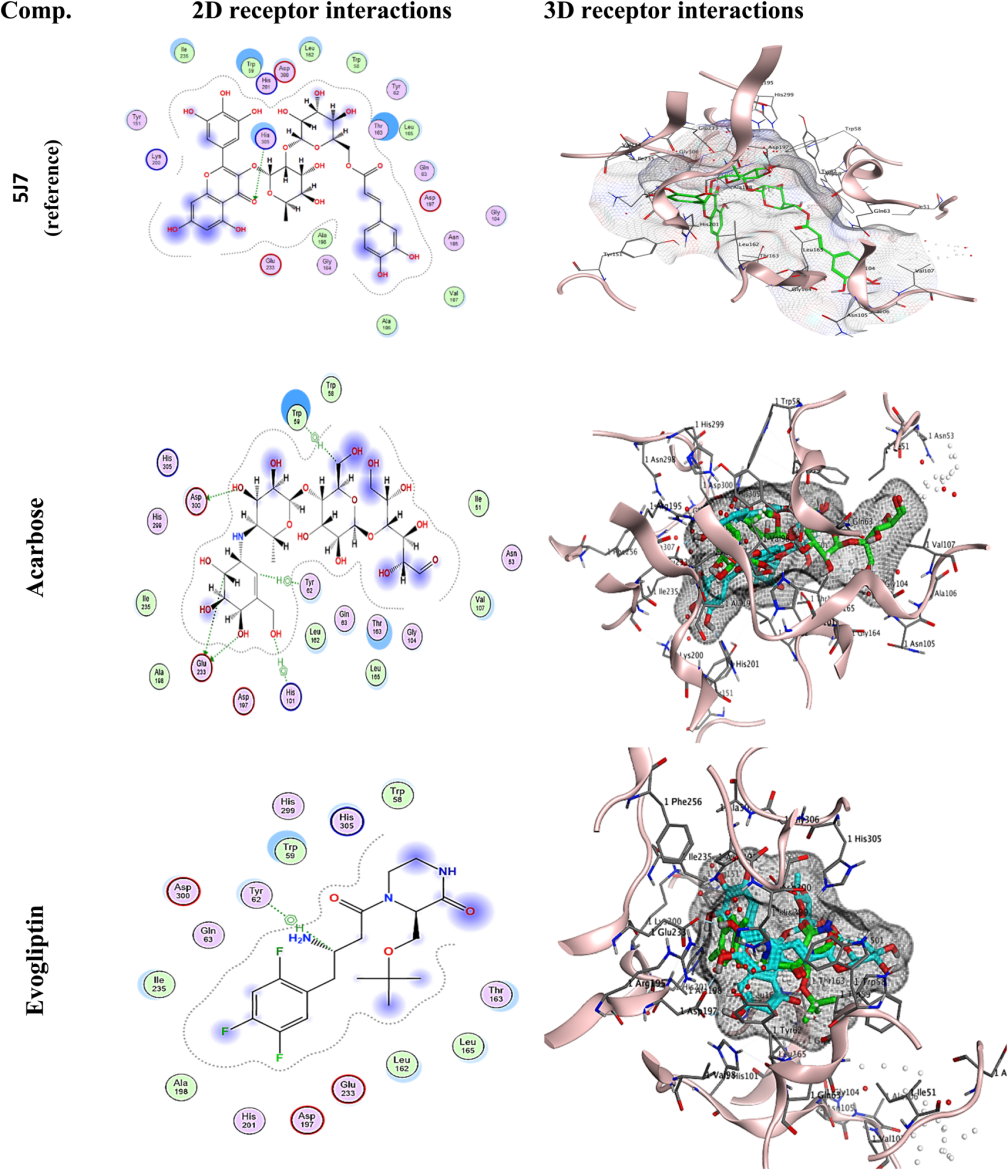
2D and 3D receptor interactions of the reference ligand [Acarbose and Evogliptin] and 5J7.

**Fig. 3:**
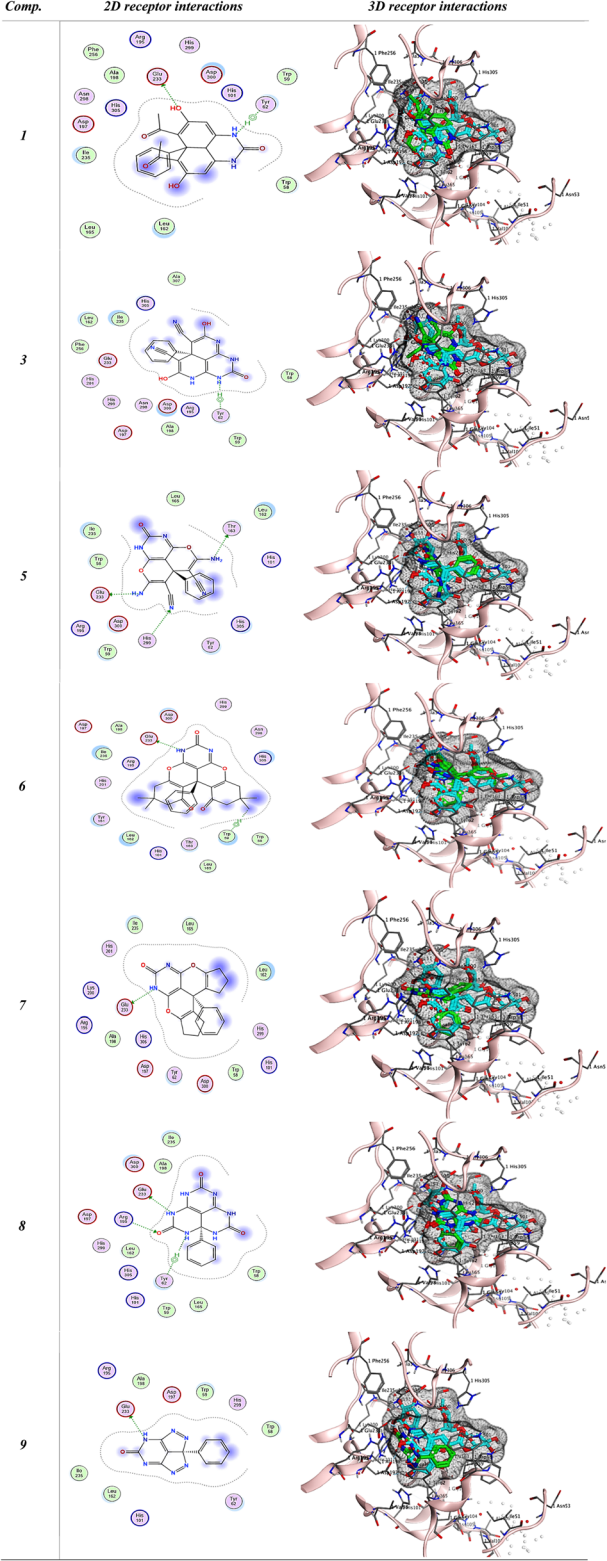
2D and 3D receptor interactions of the promising synthesized compounds.

A molecular docking analysis was conducted using MOE (ver. 2022) to confirm the interaction between synthetic compounds and targets associated with anti-diabetic illness. All 7 targets associated with anti-diabetic illness were docked separately with each medication. These compounds have extraordinarily high binding affinities for all 7 anti-diabetic targets compared to the docked ligand [Acarbose and Evogliptin] as a reference for anti-diabetic evaluation using human pancreatic alpha-amylase in complex with mini-montbretin A (PDB ID: 5E0F). Two commercial drugs [Acarbose and Evogliptin] are powerful anti-diabetic drugs that inhibit 5E0F, as shown in Table 1. Acarbose binds in a pocket dummy atom binding site, interacting with ASP 300 (A) and GLU 233 (A) with 3 hydrogen bonds and also establishing a pi-H with 5-ring TRP 59 (A), 6-ring TYR 62 (A), and 5-ring HIS 101 (A). While Evogliptin binds in a pocket dummy atom binding site, interacting with TYR 62 with pi-H. Fig. 2 depicts Acarbose and Evogliptin's binding location to 5E0F in 2D and 3D. All compounds, especially 1, 6, and 8, bind to the human pancreatic alpha-amylase in complex with mini-montbretin A (PDB ID: 5E0F) active site similarly to the native ligand, as shown in Table 1. As depicted in Table 1, target 1 was bound to the active site with docked scores of −6.047 (kcal mol^−1^). Whereas compound 6 exhibited the highest docking score of −6.6096 (kcal mol^−1^). Their re-docking poses were illustrated in Fig. 3.

To validate the docking protocol, a redocking study was performed using the native ligand (5J7) present in the crystal structure of human pancreatic α-amylase (PDB ID: 5E0F). The ligand was extracted and re-docked into the active site under the same docking conditions used for the test compounds. The obtained RMSD value for the native ligand was 1.966 Å, which is within the generally accepted threshold of ≤ 2 Å, confirming the reliability of the docking parameters.

Further validation was performed by docking two commercially available and well-characterized α-amylase inhibitors, Acarbose and Evogliptin. These inhibitors were docked under identical conditions, and their binding scores (−8.399 kcal mol^−1^ and −6.450 kcal mol^−1^, respectively), RMSD values (1.470 Å and 1.603 Å, respectively), and interaction profiles with key catalytic residues (Asp300, Glu233, Tyr62, Trp59, His101) were compared with those of the synthesized compounds. Several synthesized compounds, particularly compounds 1, 6, and 8, exhibited comparable or favorable binding affinities and similar binding modes to the reference ligands, interacting with the same critical residues in the active site (Table 3).

**Table 3:** The binding scores, RMSD values, distance, and receptor interactions of the most promising compounds (1, 3, 5, 6, 7, 8, and 9) were compared to the docked ligand [Acarbose and Evogliptin] and 5J7 as a reference for anti-diabetic evaluation

Comp	Score (kcal mol^−1^)	RMSD	Interacting residues				
			Ligand	Receptor	Interactions	Distance (Å)	*E* (kcal mol^−1^)
5J7 (reference)	−9.04865932	1.966	O 28	ND1 HIS 305 (A)	H-acceptor	2.93	−2.5
			O 62	OD1 ASP 300 (A)	H-donor	2.85	−2.0
			C 75	OE2 GLU 233 (A)	H-donor	3.41	−0.5
Acarbose	−8.39953136	1.470	O 81	OE1 GLU 233 (A)	H-donor	3.27	−1.3
			C 55	5-Ring TRP 59 (A)	H-pi	3.92	−0.6
			C 68	6-Ring TYR 62 (A)	H-pi	4.49	−0.7
			O 86	5-Ring HIS 101 (A)	H-pi	4.24	−1.0
Evogliptin	−6.45007515	1.603	C 36	6-Ring TYR 62 (A)	H-pi	4.24	−1.0
1	−6.04776144	1.365	O 11	OE2 GLU 233 (A)	H-donor	2.70	−1.7
			N 5	6-Ring TYR 62 (A)	H-pi	3.85	−0.8
3	−5.58074379	0.529	N 35	6-Ring TYR 62 (A)	H-pi	4.20	−1.8
5	−5.26979733	1.554	N 11	OE2 GLU 233 (A)	H-donor	2.98	−4.5
			N 33	OG1 THR 163 (A)	H-donor	3.04	−1.5
			N 16	NE2 HIS 299 (A)	H-acceptor	3.63	−1.0
6	−6.6096797	1.217	N 59	OE2 GLU 233 (A)	H-donor	2.99	−3.4
			C 49	5-Ring TRP 59 (A)	H-pi	4.23	−0.5
7	−5.78755617	1.989	N 43	OE1 GLU 233 (A)	H-donor	2.97	−3.9
8	−5.62768507	1.188	N 13	OE2 GLU 233 (A)	H-donor	2.90	−8.5
			O 30	NH2 ARG 195 (A)	H-acceptor	2.99	−2.4
			N 16	6-Ring TYR 62 (A)	H-pi	4.20	−0.8
9	−5.00731277	0.991	N 23	OE2 GLU 233 (A)	H-donor	2.88	−8.8

These validation steps redocking of the native ligand and comparison with known inhibitors confirm that the docking protocol is robust and the *in silico* results are credible.

### Optimization of radiochemical yield for 131-iodocomp 1

**Fig. 4:**
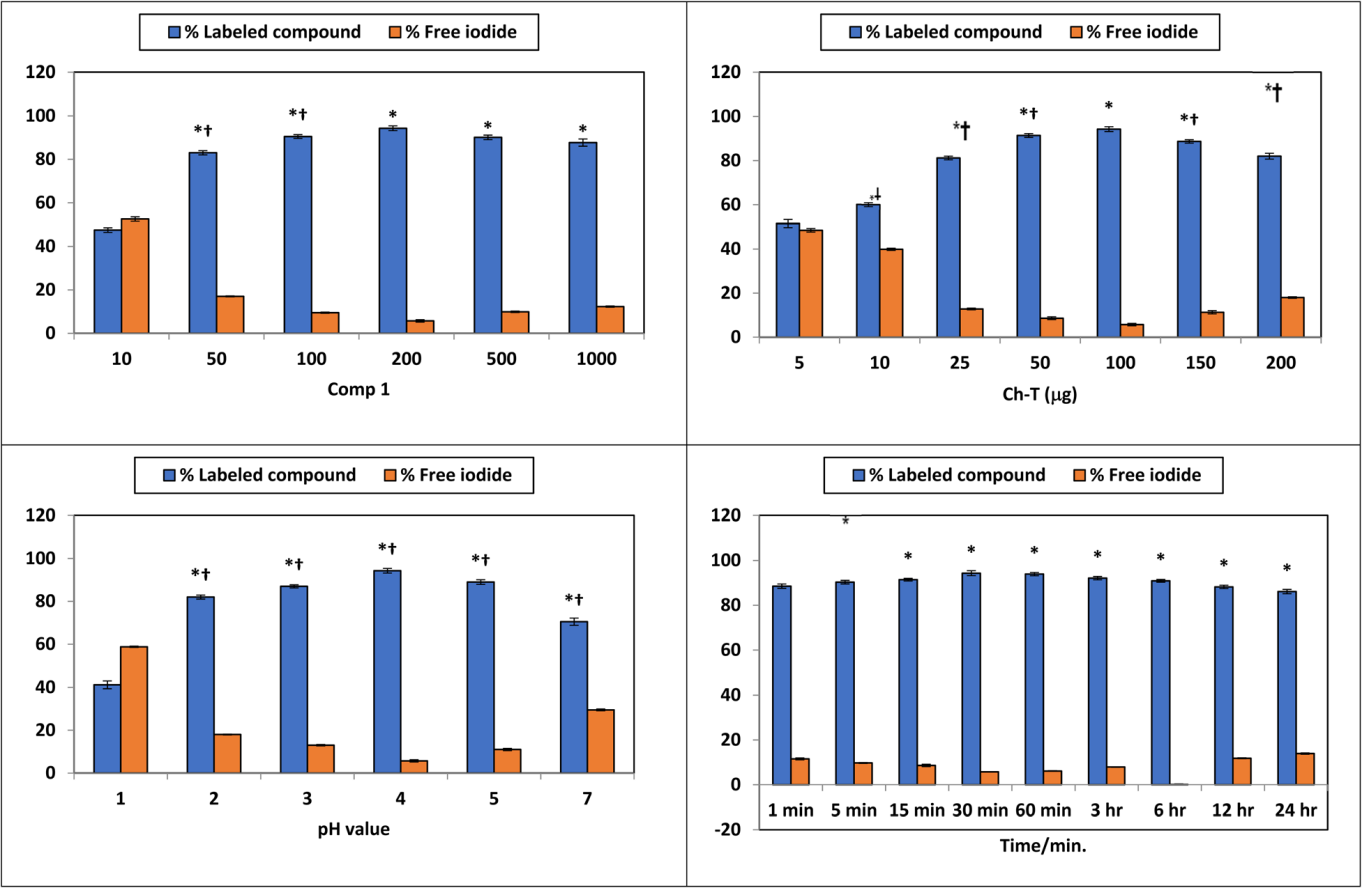
Optimization of Radiochemical Yield for 131-iodocomp 1.

To enhance the radiolabeling efficiency of 131-iodocomp 1, the experimental conditions were systematically adjusted, including the concentrations of comp 1 and CH-T, as well as the pH, reaction duration, and temperature (Fig. 4). The highest radiochemical yield (RCY) achieved was 94.3 ± 1.1%, as measured by paper chromatography. This optimal result was obtained when the reaction used 200 μg ml^−1^ of comp. 1 and 100 μg of ChT, conducted at pH 4 for 30 minutes at room temperature (25 °C), Fig. 4.

### Bio-evaluation

#### *In vitro* α-Amylase biological evaluation

**Fig. 5:**
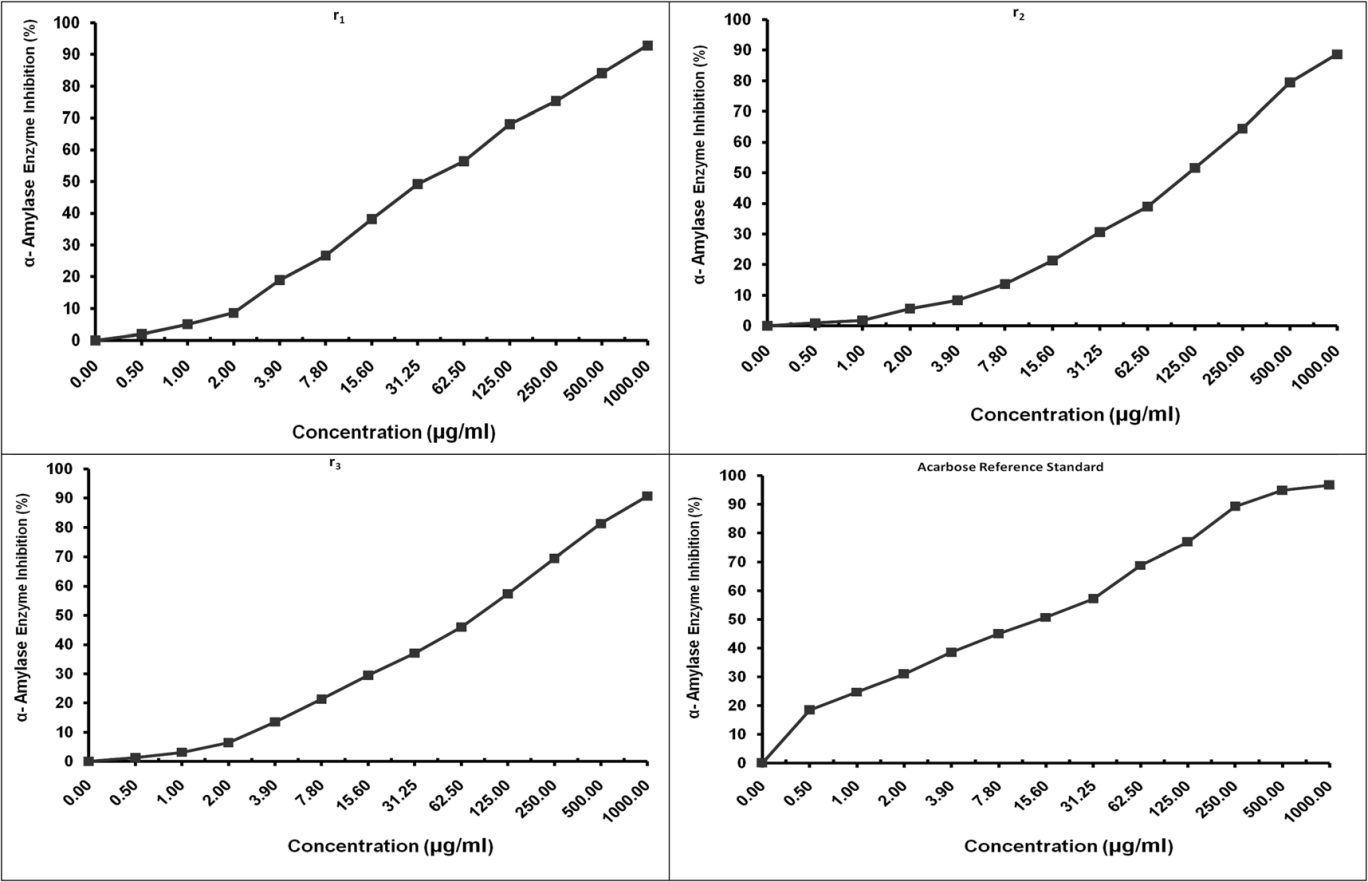
Elucidate the amylase inhibitory effects of compounds 1, 6, 8, and standard acarbose, respectively, showing the superiority of compound 1 (IC_50_ = 34.60 ± 1.43 μg ml^−1^) relative to compounds 6 and 8.

The α-amylase inhibition activity was estimated for compounds 1, 6, and 8, with acarbose as the standard. In Fig. 5, all tested compounds showed inhibitory potential to different degrees. Compound 1 exhibited the highest inhibiting activity, with an IC_50_ of 34.60 ± 1.43 μg ml^−1^. This result agrees with the compound's structural characteristics, forming favorable binding site activity of α-amylase, as the *in silico* findings show.

Our pyrimidine scaffold (*e.g.*, Compound 1) offers distinct advantages over recent pyrimidine-based α-amylase inhibitors:

Our pyrimidine scaffold exhibits a distinctive structural profile and functional versatility compared to previously reported pyrimidine-based α-amylase inhibitors. Unlike the thiazolopyrimidine carboxylates reported by Batool *et al.* (2016),^50^ which lack dual polar substituents, our design integrates both acetyl and hydroxyl moieties, enabling enhanced hydrogen bonding with Glu233 and Tyr62 in the α-amylase active site (docking score −6.05 kcal mol^−1^*vs.* −5.29 kcal mol^−1^). Moreover, the dual theranostic potential combining α-amylase inhibition with radiolabeling for imaging represents a rare approach, as prior works such as Chigurupati *et al.* (2021) focused solely on enzymatic inhibition.^20^ The TiO_2_-nanocatalyzed synthesis also achieved a significantly higher yield (98% in 6 h) compared to conventional protocols (*e.g.*, 58% in 15 h, Hassaballah *et al.*, 2024), underscoring its green and efficient nature.^51^

#### *In Vivo* biodistribution study

**Fig. 6:**
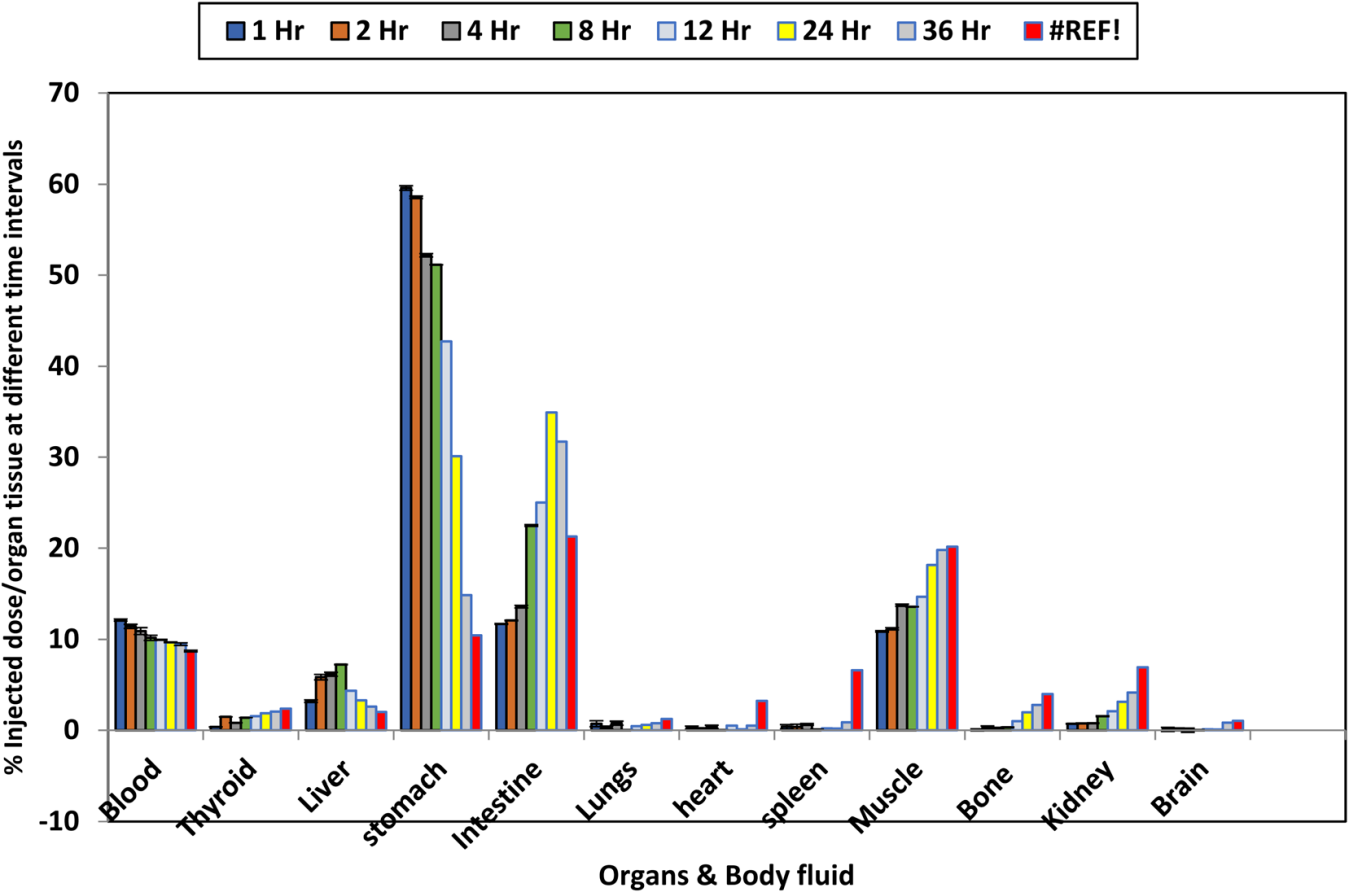
elucidates the organs uptake as % injected dose/organ tissue at different times was expressed as X±S.E.

The *in vivo* behavior of the highest amylase inhibitor compound 1 was studied using [^131^I]-labeled mice. Radioactivity levels were measured in major organs at different time points (1 to 36 hours post-injection), and results were expressed as % injected dose per gram of tissue, Fig. 6. Following administration, a marked initial accumulation was seen in the stomach (59.58% ID per g) and intestine (11.70% ID per g) at the 1 hour post-injection. Gastric levels decreased slowly, while intestinal uptake increased, reaching a maximum at 16 hours (34.93% ID per g). This distribution profile is indicative of gastrointestinal slow release, which is potentially attributed to the compound's α-amylase affinity and possibly species differences in gastric retention in mice. These findings are comparable with compounds that block carbohydrate digestion enzymes, such as acarbose, and may indicate slow intestinal clearance (enterohepatic circulation) or mucosal interaction.^52^ The stable blood levels from 12.13% ID per g at 1 hour to 8.72% ID per g at 36 hours indicate evidence of low systemic clearance, possibly enhancing the compound's availability for therapeutic action through re-excretion to the intestine. There was a continuous increase in muscle uptake with time, reaching 20.15% ID per g at 36 hours, indicating progressive peripheral uptake. This muscle uptake retention highlights a potential link to muscle-related side effects (*e.g.*, weakness, fatigue) reported with acarbose, though the mechanism is unclear. It is possible that amylase-binding-related compounds like this may be retained in muscle tissue, either through passive diffusion or transport mechanisms related to glucose metabolism or unknown mechanisms.^53^ Liver uptake reaches maximum at 8 hours, indicating early hepatic metabolism. This uptake is similar for compounds showing phase I/II metabolism. Kidney uptake increased continuously, indicating some renal clearance as a possible primary elimination route. The slow increase in thyroid uptake (2.38% ID per g at 36 h), bone, and spleen radioactivity may highlight *in vivo* deiodination, a known equilibrium of radioiodinated tracers, leading to radio uptake of free [^131^I] in iodine-avid tissues. Such deiodination limits imaging specificity but could be minimized by modifying the compound to resist enzymatic degradation or by using alternative radionuclides with greater *in vivo* stability.^54^ Accordingly, Compound 1 demonstrated the highest α-amylase inhibition *in vitro* and a favorable biodistribution profile, particularly with regard to prolonged intestinal residence. The slow gastric clearance and prominent intestinal and muscle uptake highlight its potential as a gastrointestinal-targeted antidiabetic agent, as well as a radiotracer for GI imaging probes. The continuous muscle accumulation observed may give a new glimpse of the muscle-related side effects that were found with amylase inhibitors. Further studies are needed to find out the mechanisms attributed to side effects and to explore the clinical perspective of this compound as both a therapeutic and diagnostic agent.

The *in vivo* biodistribution profile of ^131^I–Compound 1 demonstrated marked gastrointestinal (GIT) specificity and stability when compared with control data. Free ^131^I is known to accumulate predominantly in the thyroid due to iodide trapping; however, ^131^I–Compound 1 exhibited minimal thyroid uptake (<3% ID per g), confirming *in vivo* stability and reduced deiodination.^55^ Notably, stomach retention was significantly higher (59.58 ± 3.2% ID per g at 1 h) than literature-reported values for the α-amylase inhibitor acarbose (∼15% ID per g at 4 h).^52^ Sustained intestinal accumulation (34.93% ID per g at 16 h) was approximately threefold greater than in muscle, indicating selective GIT targeting. This slow intestinal clearance aligns with prolonged luminal α-amylase inhibition, which is advantageous for maintaining therapeutic concentrations at the primary enzymatic site while minimizing systemic exposure.

The pronounced accumulation of the radiolabeled pyrimidine derivative in the stomach (59.58% ID per g) and intestine (34.93% ID per g) is consistent with the luminal localization of α-amylase, a digestive enzyme predominantly secreted into the gastrointestinal tract.^21^ This preferential uptake suggests that the compound can achieve high local concentrations at the primary site of enzymatic activity, potentially enhancing inhibitory efficacy while minimizing systemic exposure. Such targeted distribution supports its theranostic potential, enabling simultaneous enzyme inhibition and imaging within the gastrointestinal environment.

## Conclusion

The findings of this study demonstrate the successful development of a pyrimidine-based compound with dual therapeutic and diagnostic potential for diabetes management. Several synthesized derivatives exhibited strong *in silico* binding affinities toward α-amylase, with compound 1 emerging as the most promising candidate. Guided by molecular docking and *in vitro* enzyme inhibition assays, compound 1 was radiolabeled with iodine-131, and key parameters, including ligand concentration, pH, and reaction time, were optimized to achieve high radiochemical yield and stability. Notably, compound 1 displayed both potent α-amylase inhibitory activity and a favorable pharmacokinetic profile. Its high accumulation in the gastrointestinal tract underscores its potential role in regulating postprandial glucose levels. Moreover, its gradual uptake in muscle tissue suggests possible systemic effects that may provide insight into muscle-related outcomes associated with α-amylase inhibitors. The compound's stable radiolabeling and prolonged tissue retention further support its utility as a potential molecular imaging agent for gastrointestinal applications, alongside its therapeutic promise. Nevertheless, additional studies are warranted to refine its molecular structure, improve specificity, and minimize off-target distribution.

## Ethical statement

All animal procedures were performed in accordance with the CIOMS and ICLAS International Guiding Principles for Biomedical Research Involving Animals (2012) and approved by the Research Ethics Committee of the National Center for Radiation Research and Technology (REC-NCRRT), Egyptian Atomic Energy Authority, Cairo, Egypt (Protocol No.: P/99A/24).

## Author contributions

Doaa A. Elsayed: Writing – review, editing, methodology, investigation, formal analysis, data curation, editing computational study & conceptualization. Wael Shehta: Writing—review, editing, methodology, investigation, formal analysis, data curation & conceptualization. S. El-Kalyoubi: Writing—review, editing, methodology, investigation, formal analysis, data curation & conceptualization. Adli Selim: Writing – review, editing, methodology, investigation, formal analysis, data curation & conceptualization for biology, and optimization of radiochemical yield for 131-iodo. Mohamed G. Assy: Visualization, supervision, project administration, investigation, formal analysis, data curation & conceptualization. Omar Metwally: Writing—review, editing, methodology, investigation, formal analysis, data curation & conceptualization. Ahmed A. Al-Kubaisi: Writing—review & funding acquisition. Sameer A. Awad: Writing—review & funding acquisition. F, Marzook: Writing – review, editing, methodology, investigation, formal analysis, data curation & conceptualization for biology, and optimization of radiochemical yield for 131-iodo.

## Conflicts of interest

The authors declare that they have no known competing financial interests or personal relationships that could have appeared to influence the work reported in this paper.

## Supplementary Material

RA-015-D5RA04955E-s001

## Data Availability

The data supporting this article have been included as part of the SI. The SI includes detailed experimental procedures, full characterization data of the synthesized compounds (^1^H NMR, ^13^C NMR, IR spectra). See DOI: https://doi.org/10.1039/d5ra04955e.
