# Supplementary material for: Synthesis and radiolabelled evaluation of novel pyrimidine derivatives as dual α-amylase inhibitors and GIT-targeted molecular imaging probe
Source: RSC Adv. 2025 Aug 21;15(36):29544–59. doi: 10.1039/d5ra04955e (PMC12376908; doi:10.1039/d5ra04955e)
Supplement: RA-015-D5RA04955E-s001 [file RA-015-D5RA04955E-s001.pdf]

Supplementary Information (SI) for RSC Advances.
This journal is © The Royal Society of Chemistry 2025

# SPECTRAL ANALYSIS

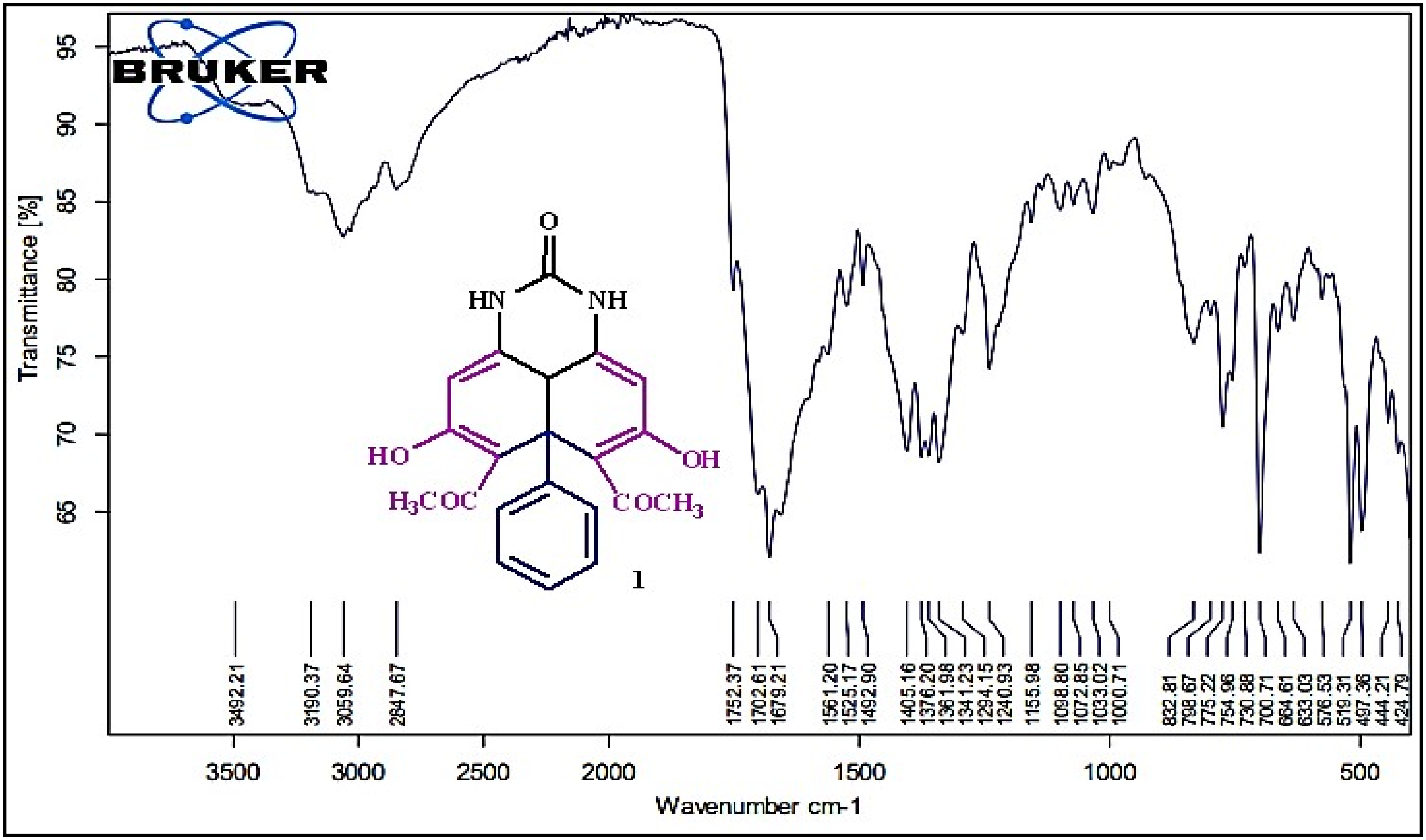


**Fig. 1**: **IR Spectrum of compound 1**

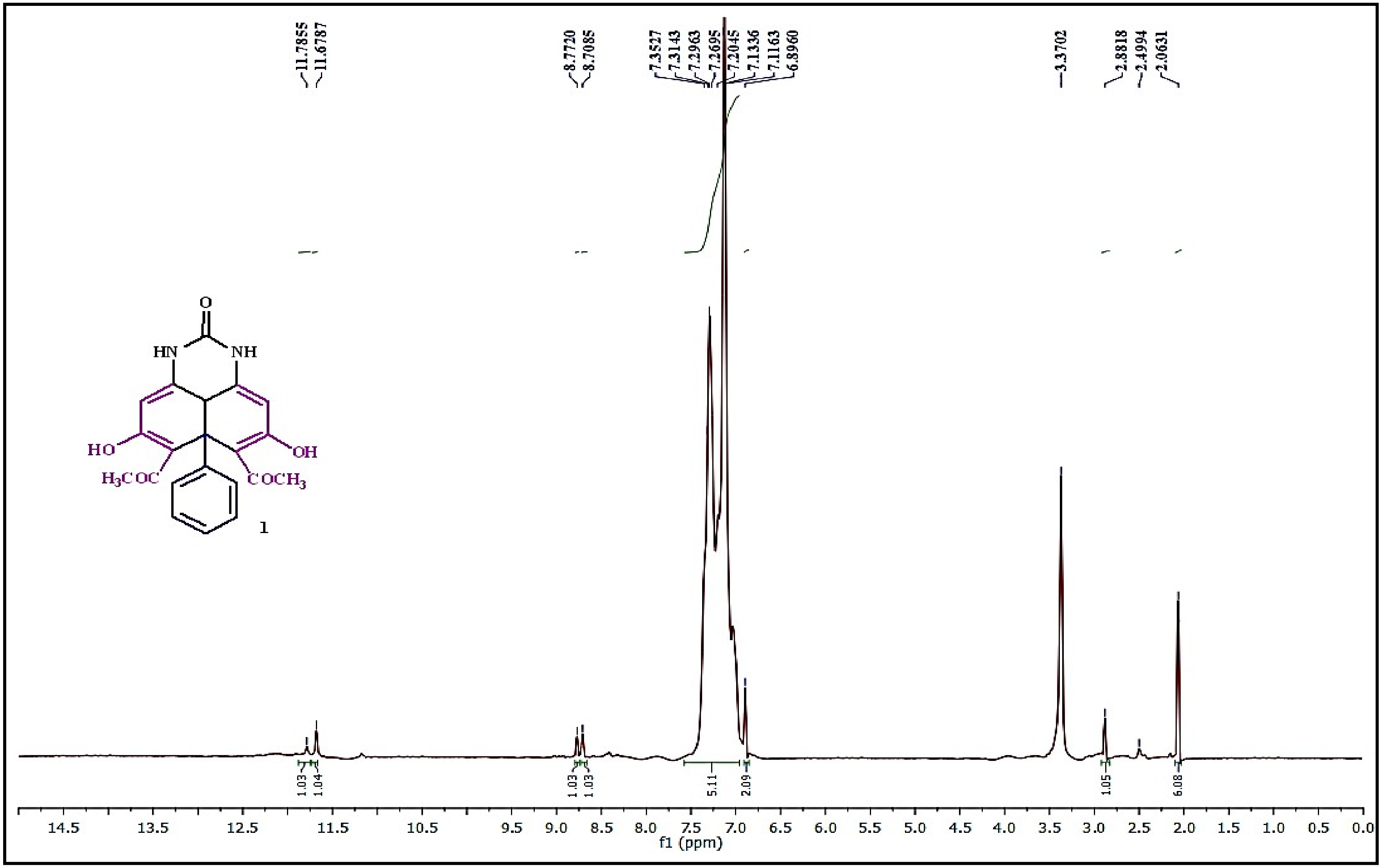


**Fig. 2**: **$^{1}$H-NMR Spectrum of compound 1**

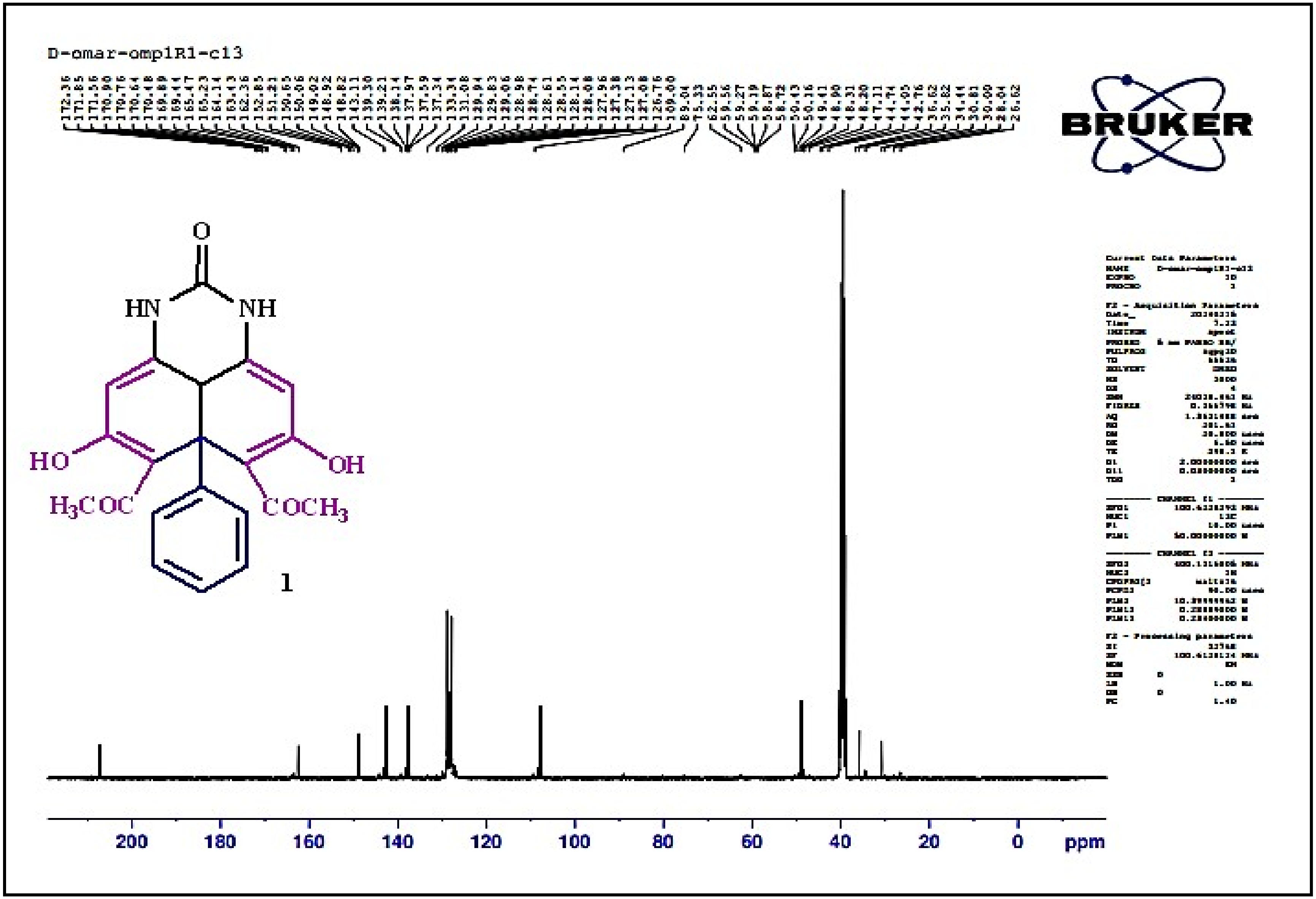


**Fig. 3**: $^{13}$C-NMR Spectrum of compound 1

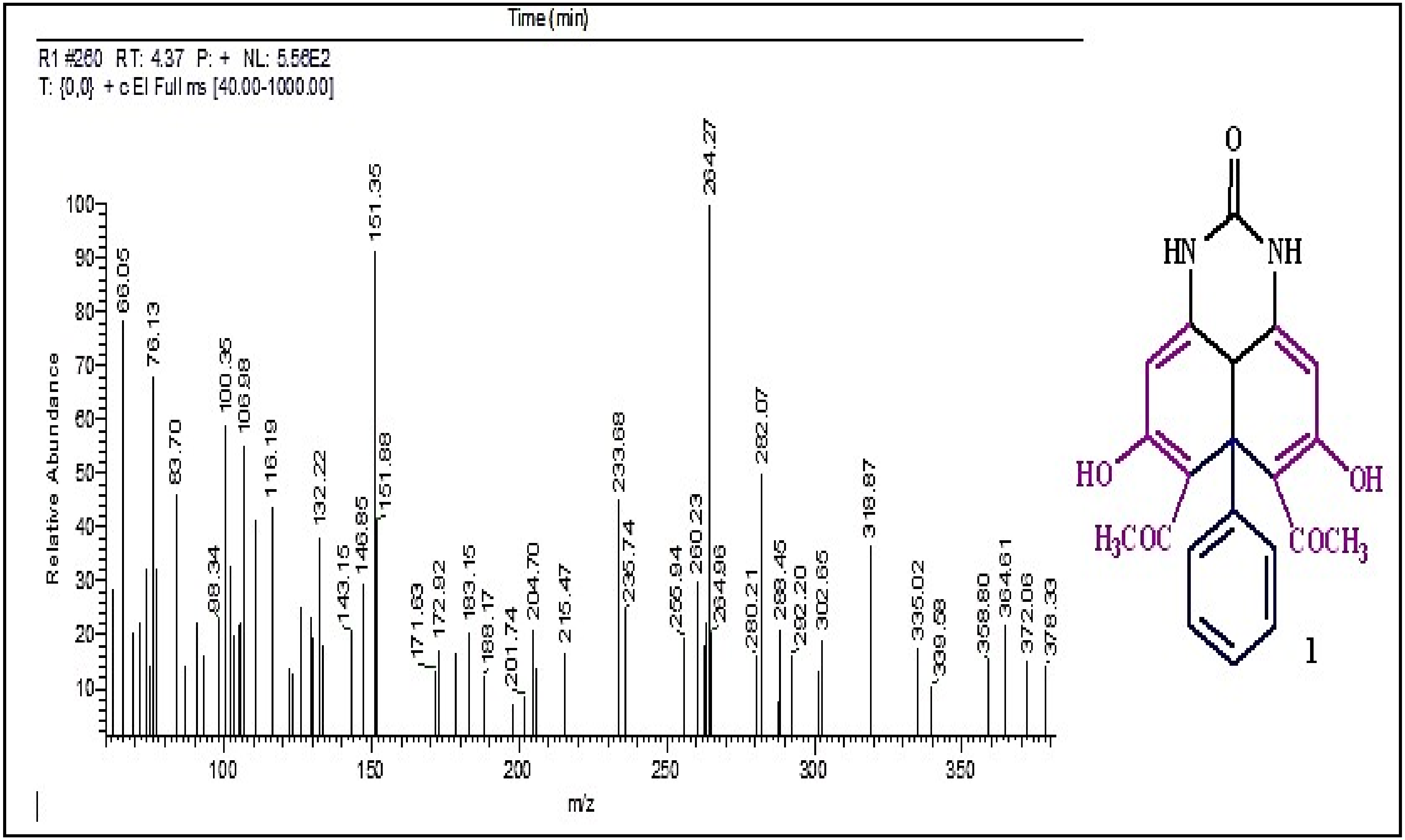


**Fig. 4**: **mass Spectroscopy of compound 1**

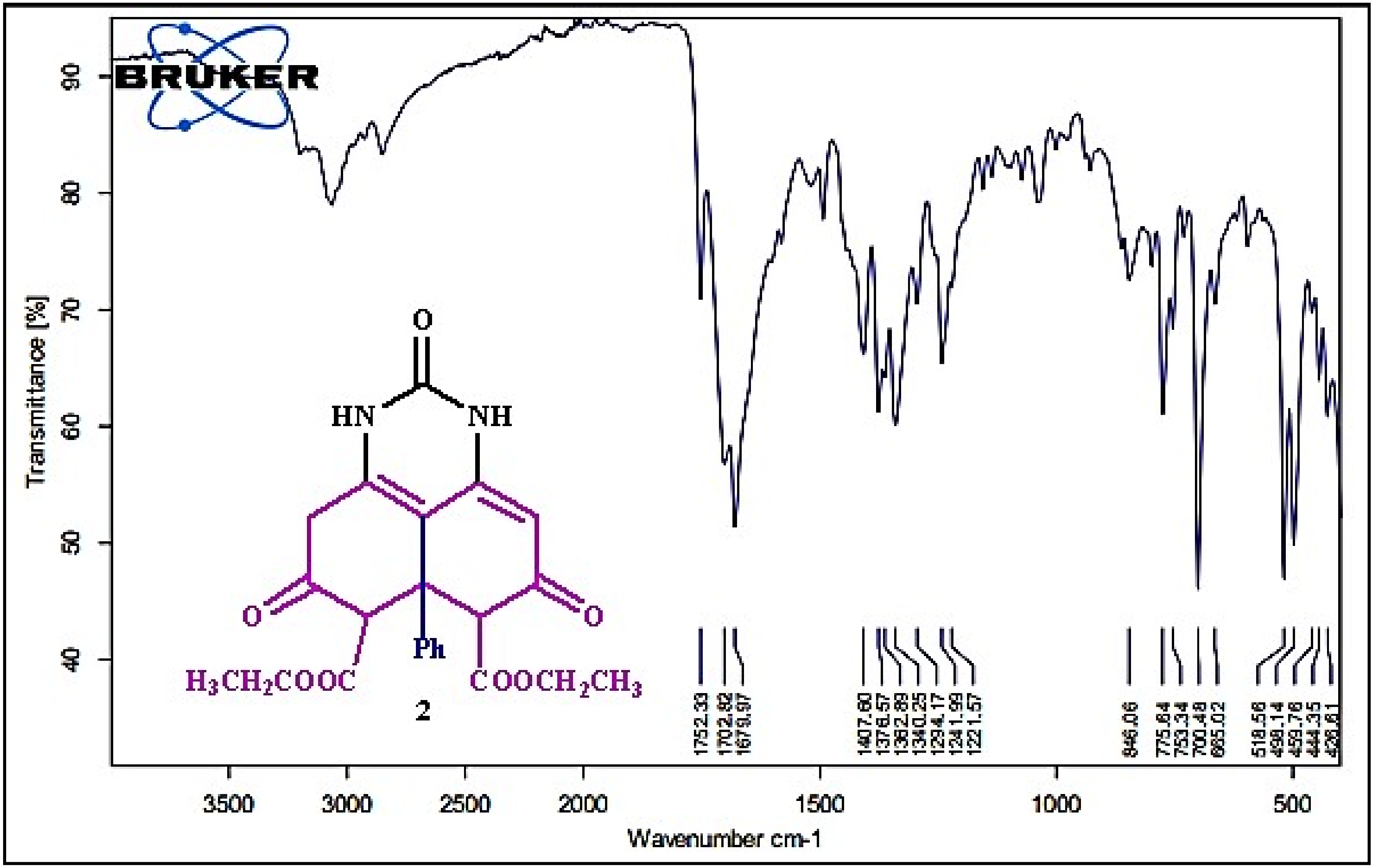


**Fig. 5**: **IR Spectrum of compound 2**

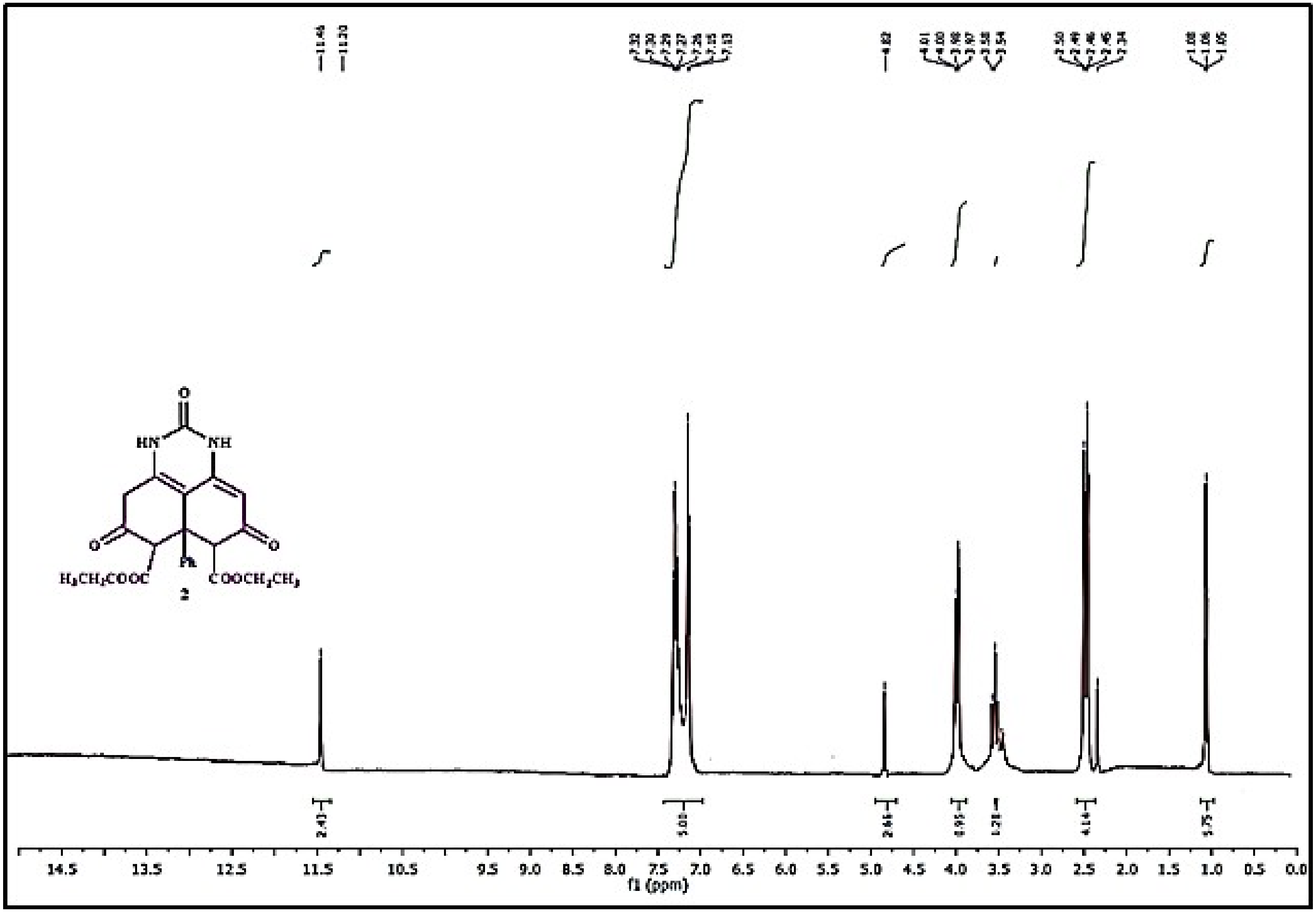

**Fig. 6**: $^1$H-NMR Spectrum of compound 2

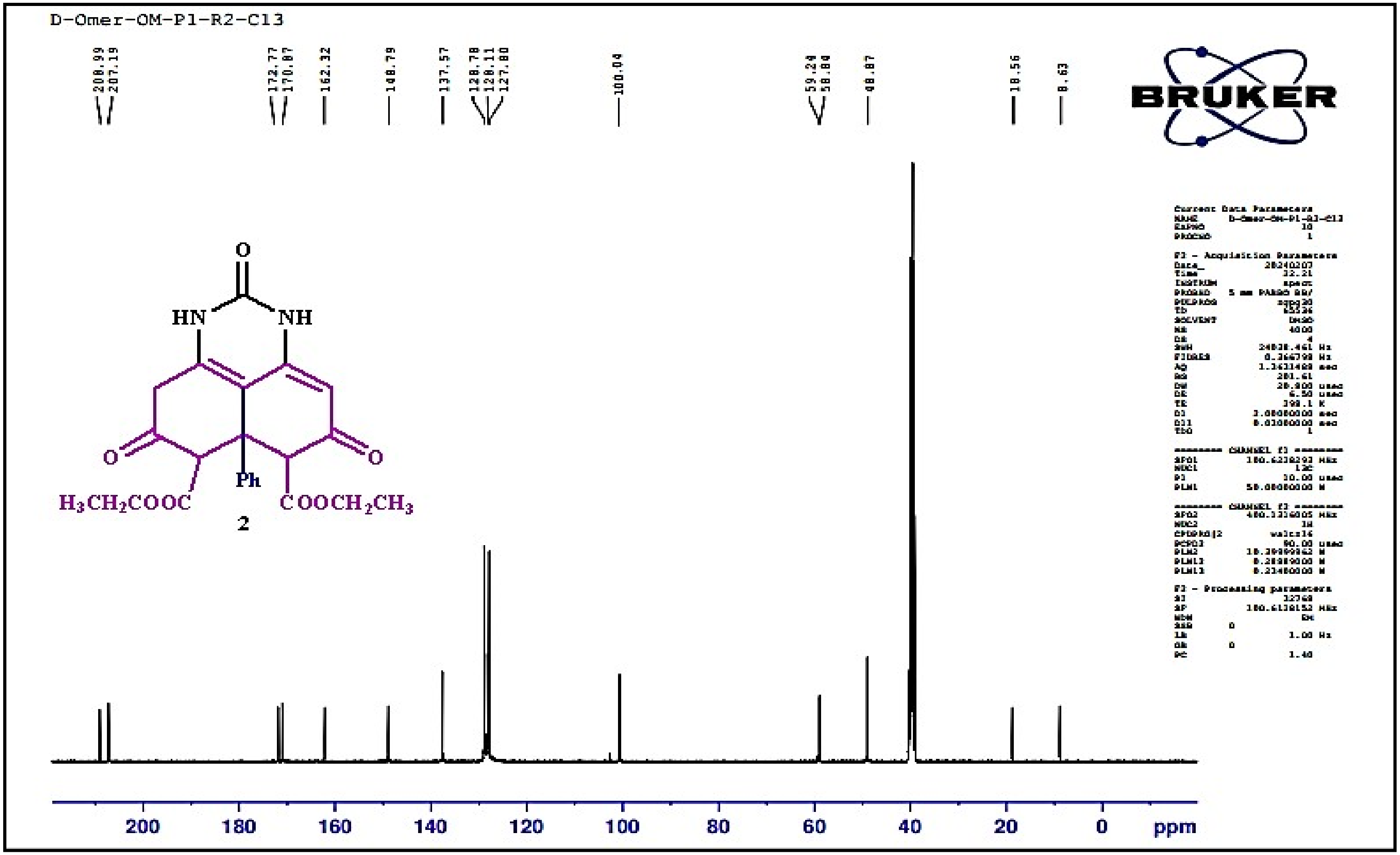


**Fig. 7**: **$^{13}$C-NMR Spectrum of compound 2**

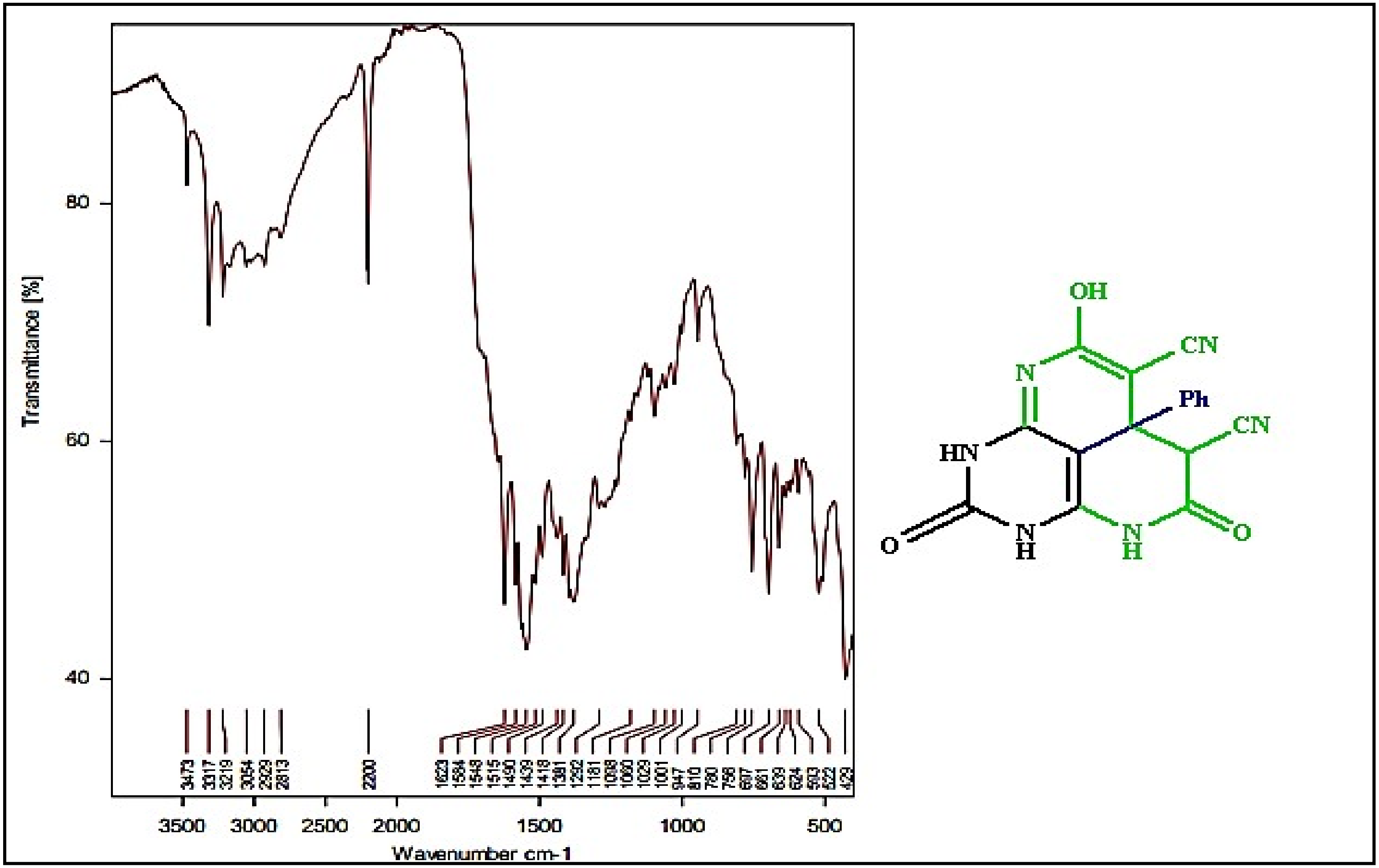


**Fig. 8**: **IR Spectrum of compound 3**

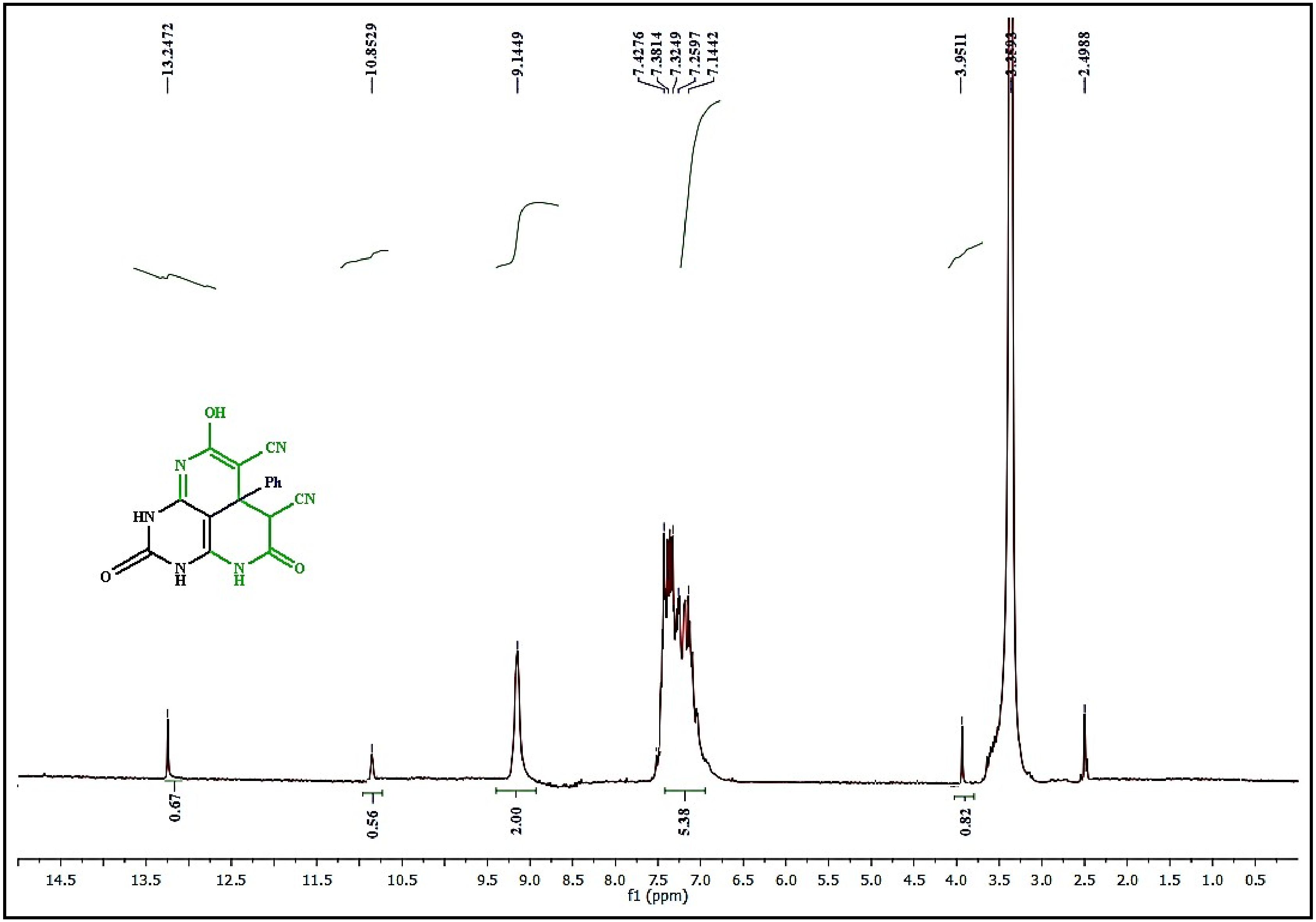


**Fig. 9**: $^{1}$H-NMR Spectrum of compound 3

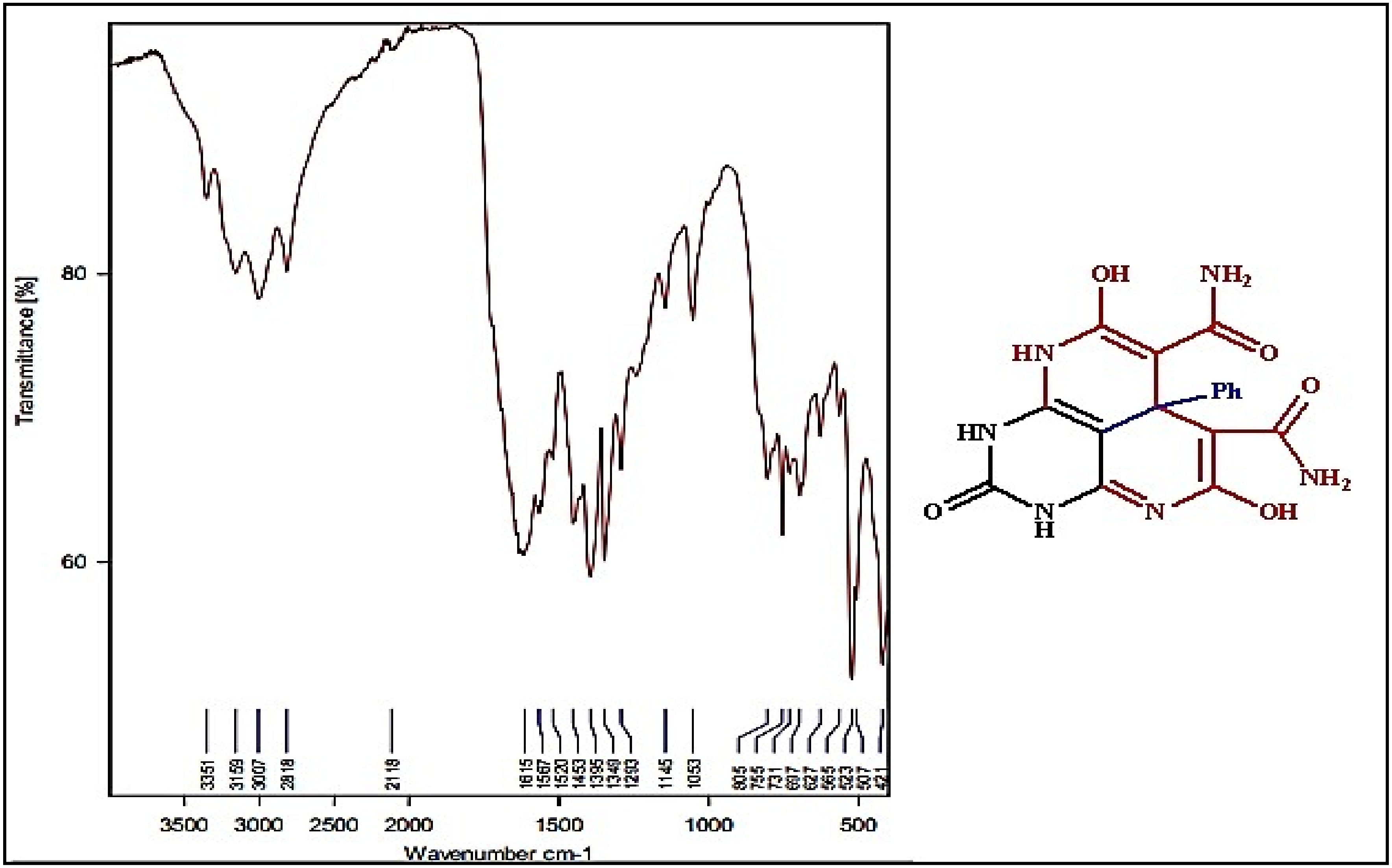


**Fig. 10**: **IR Spectrum of compound 4**

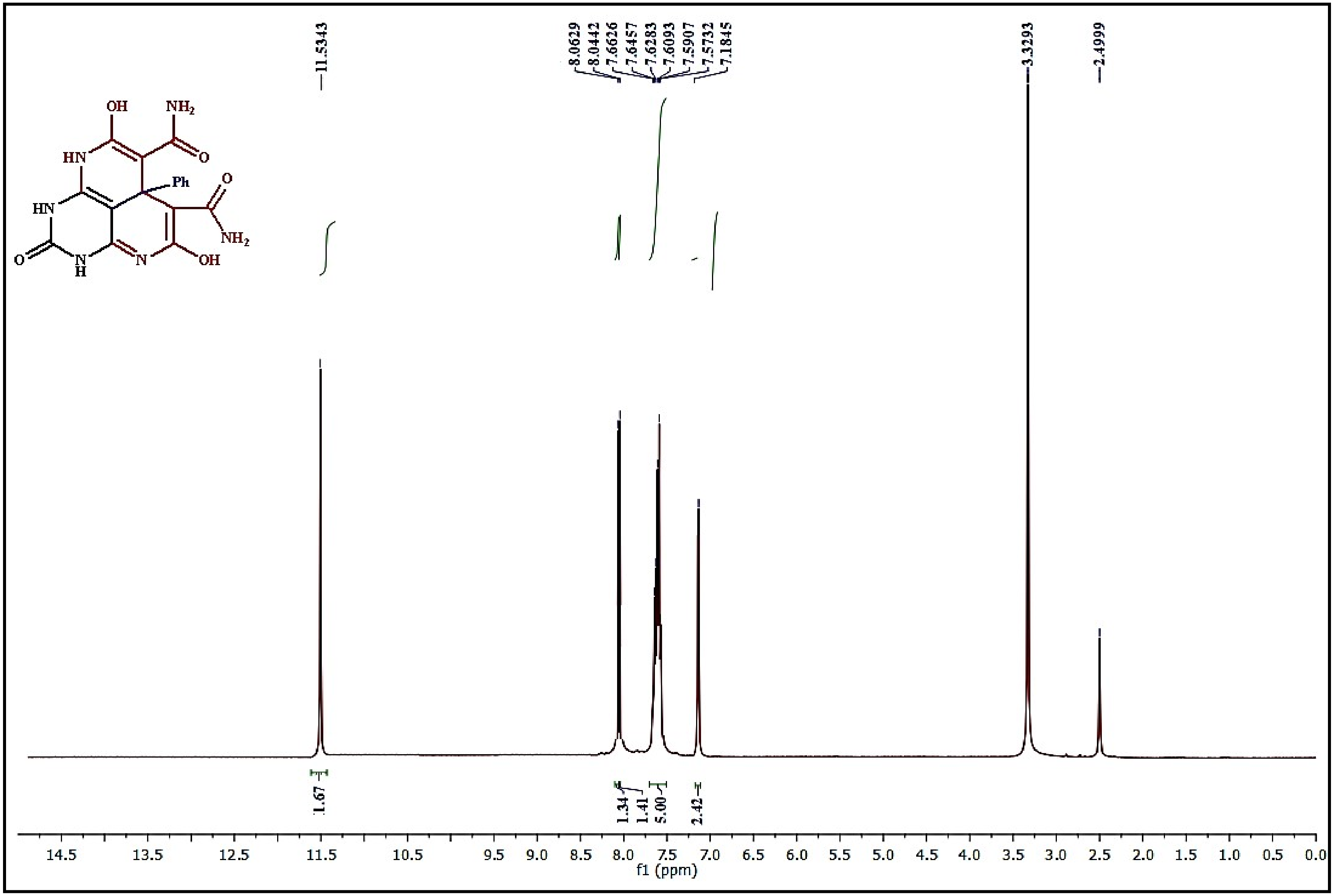


**Fig. 11**: $^{1}$H-NMR Spectrum of compound 4

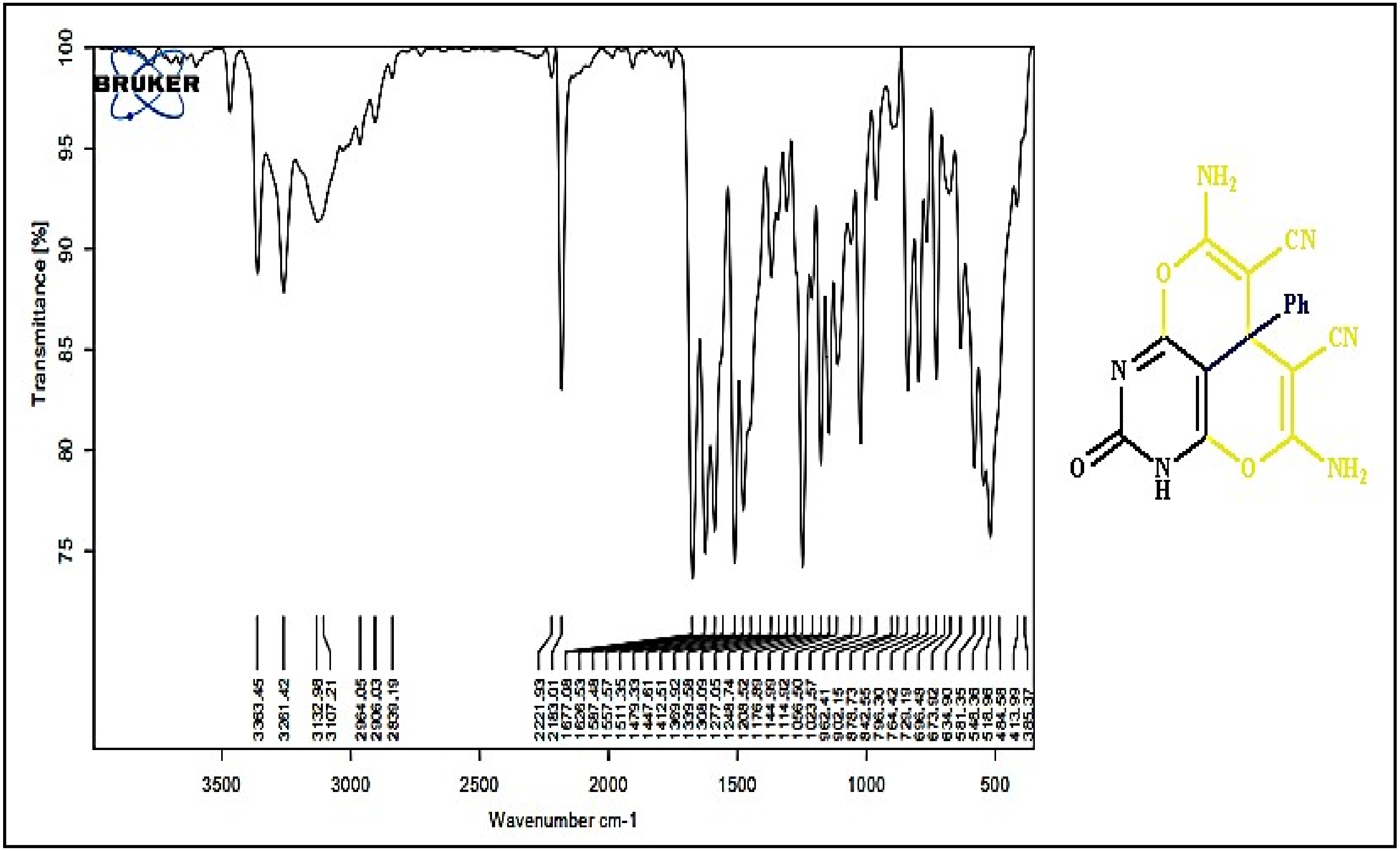


**Fig. 12**: **IR Spectrum of compound 5**

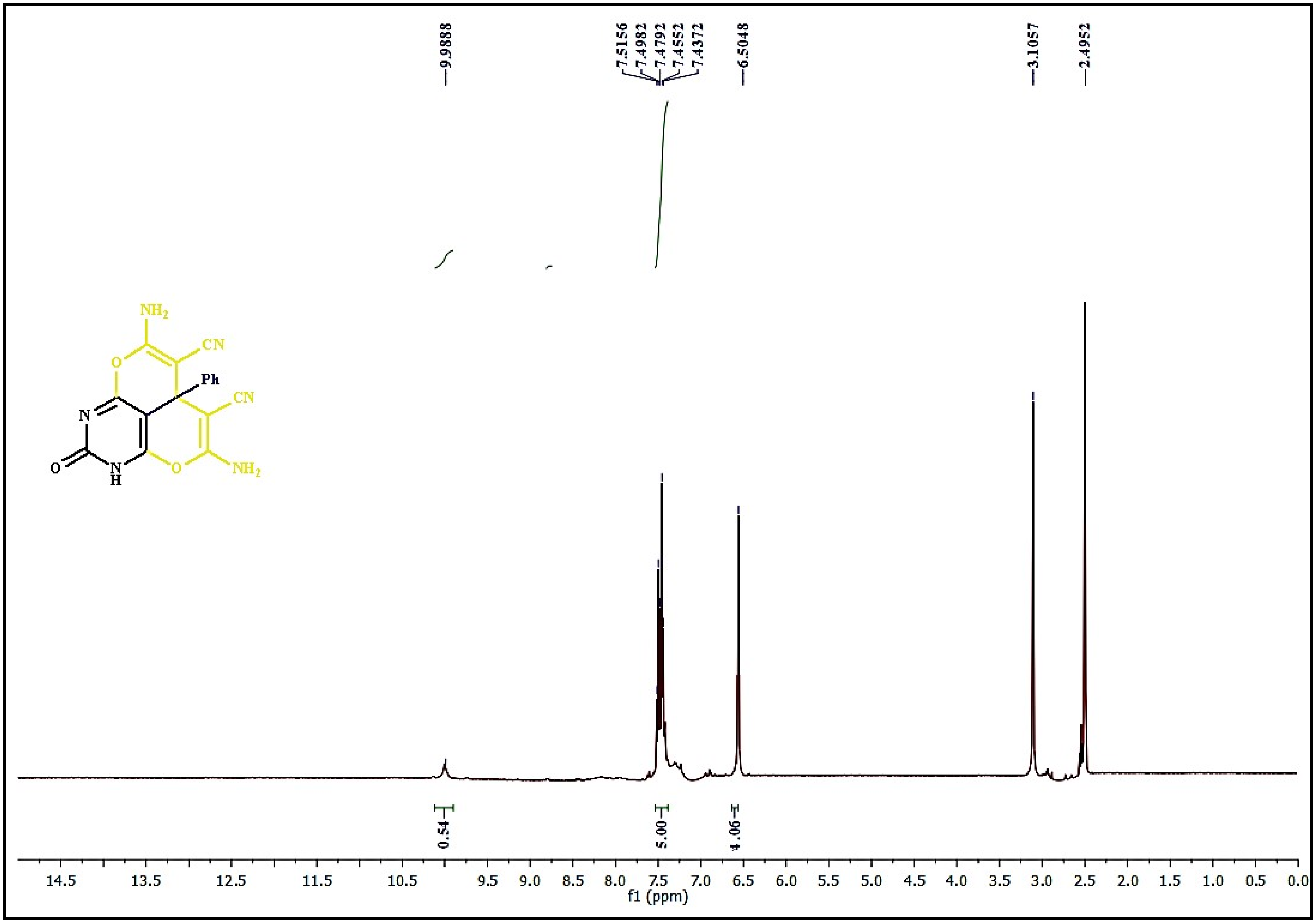


**Fig. 13**: $^{1}$H-NMR Spectrum of compound 5

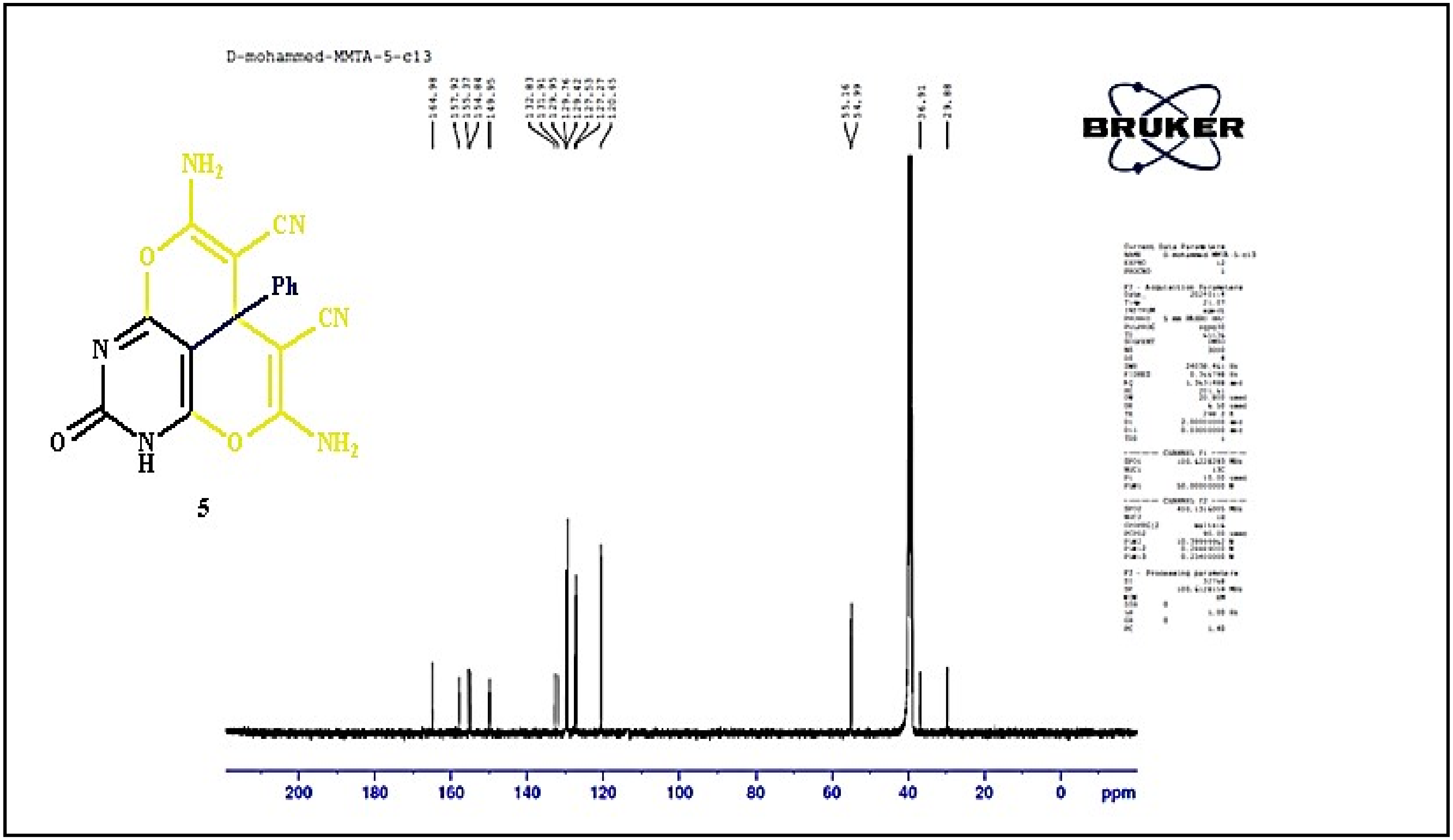


**Fig. 14**: **$^{13}$C-NMR Spectrum of compound 5**

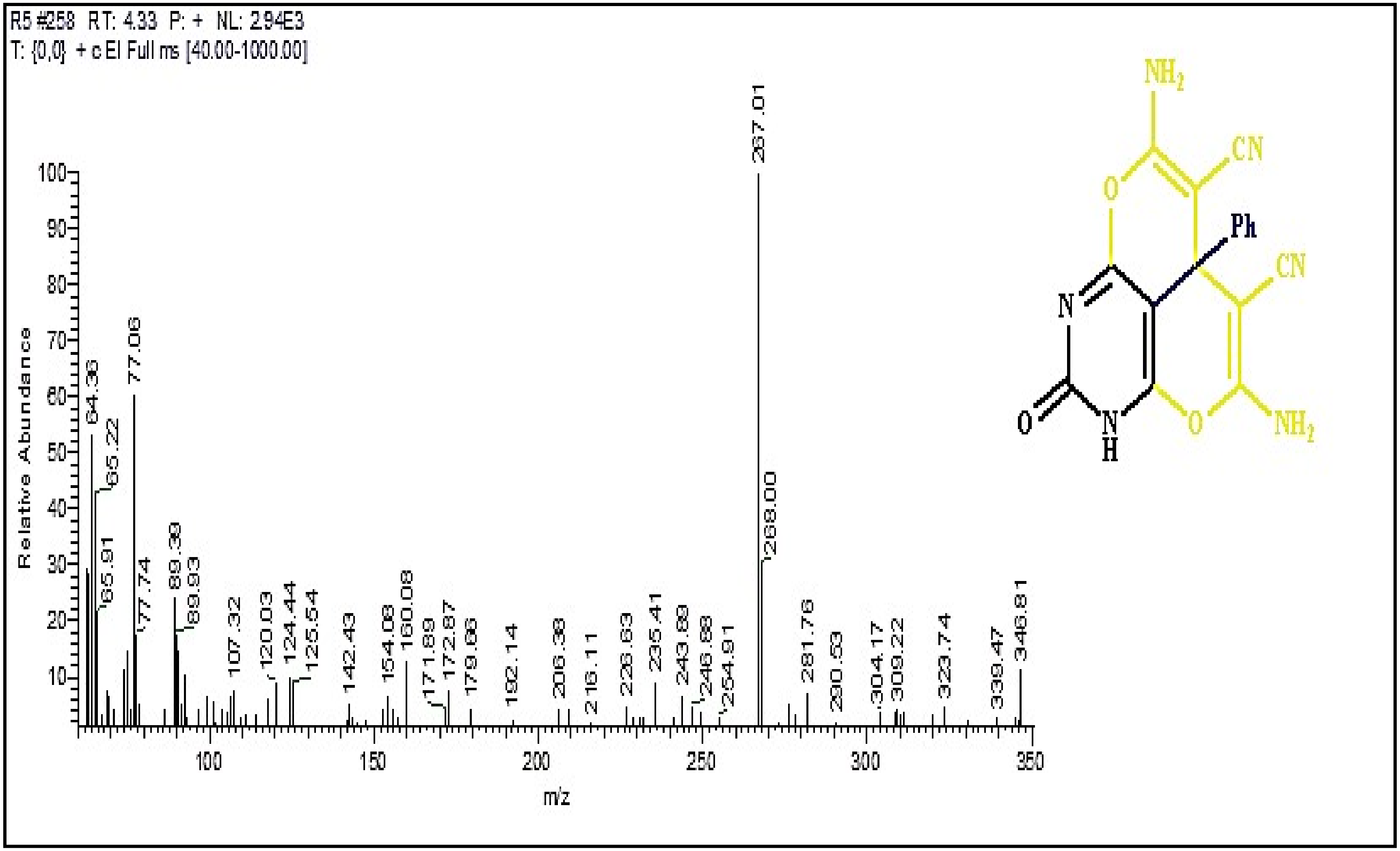


**Fig. 15**: **mass Spectroscopy of compound 5**

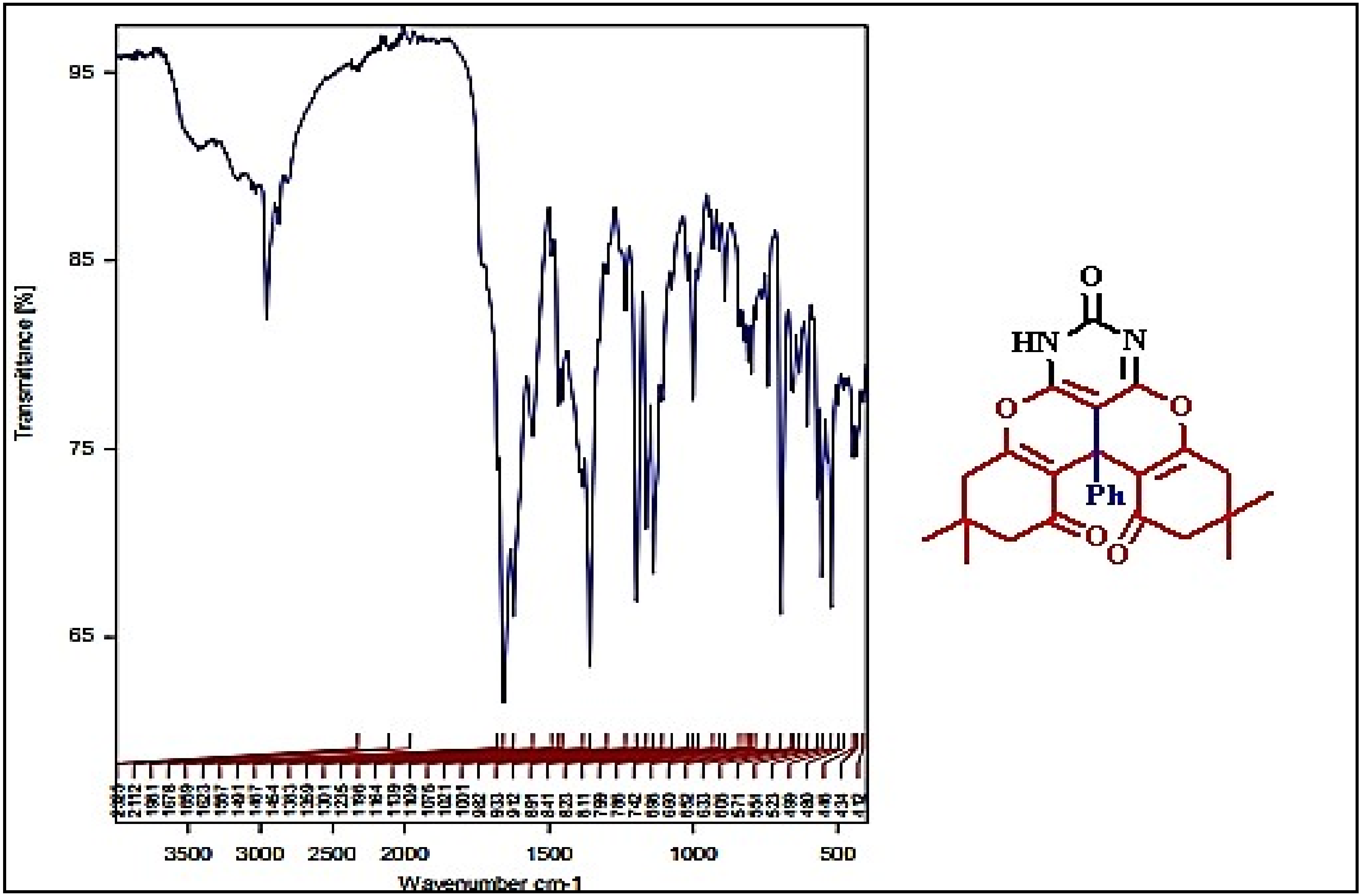


**Fig. 16**: **IR Spectrum of compound 6**

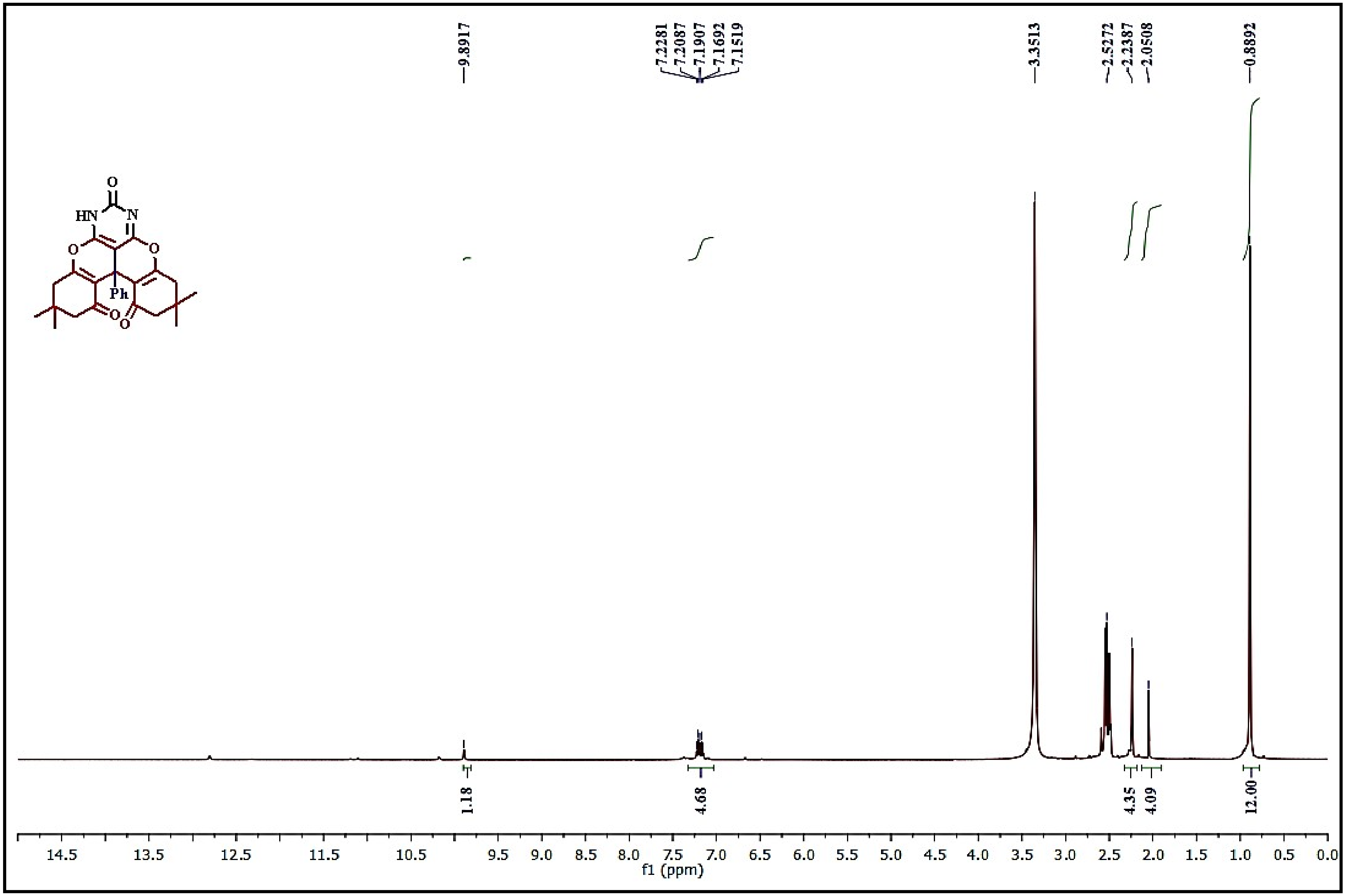


**Fig. 17**: $^{1}$H-NMR Spectrum of compound 6

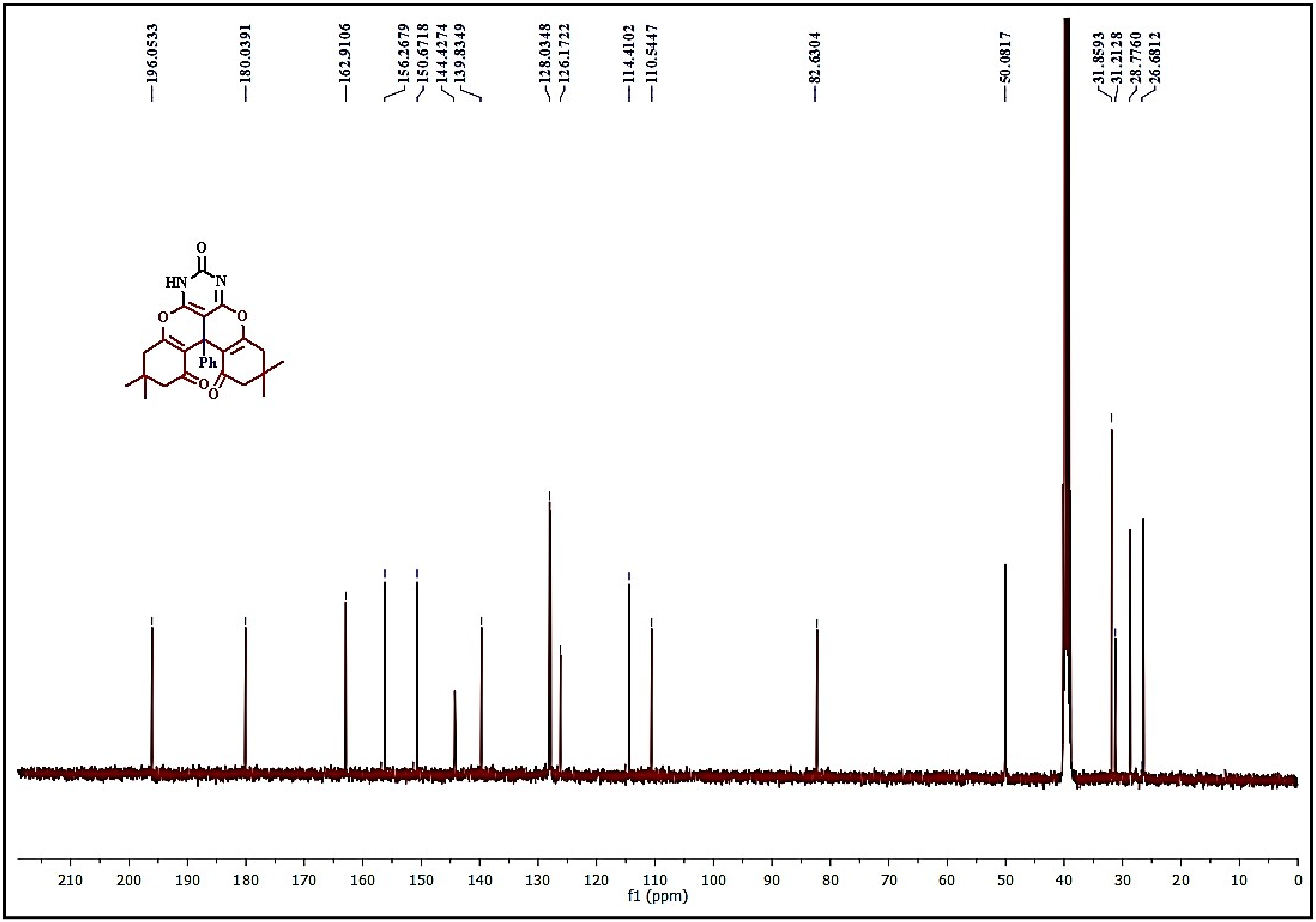


**Fig. 18**: **$^{13}$C-NMR Spectrum of compound 6**

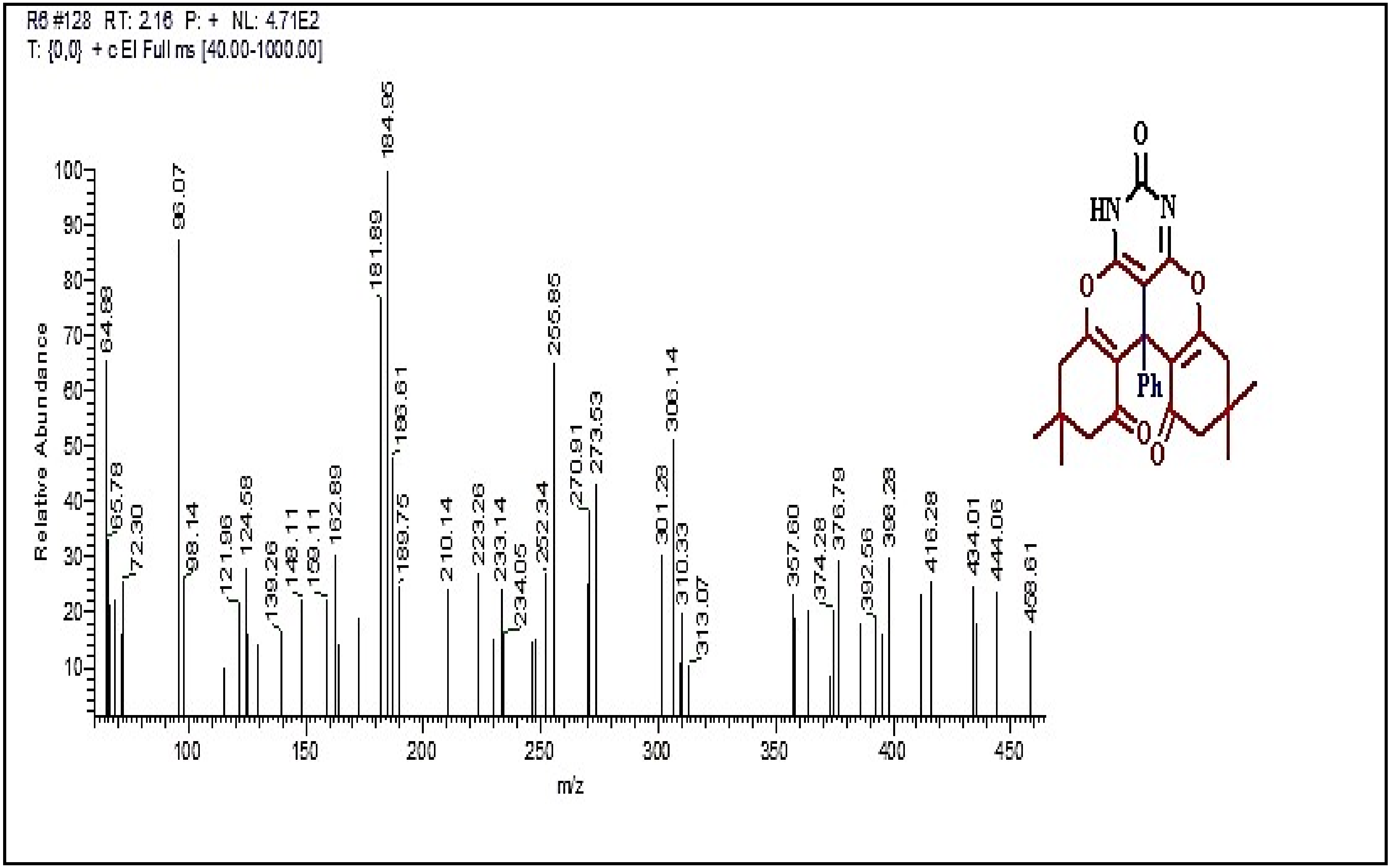


**Fig. 19**: **mass Spectroscopy of compound 6**

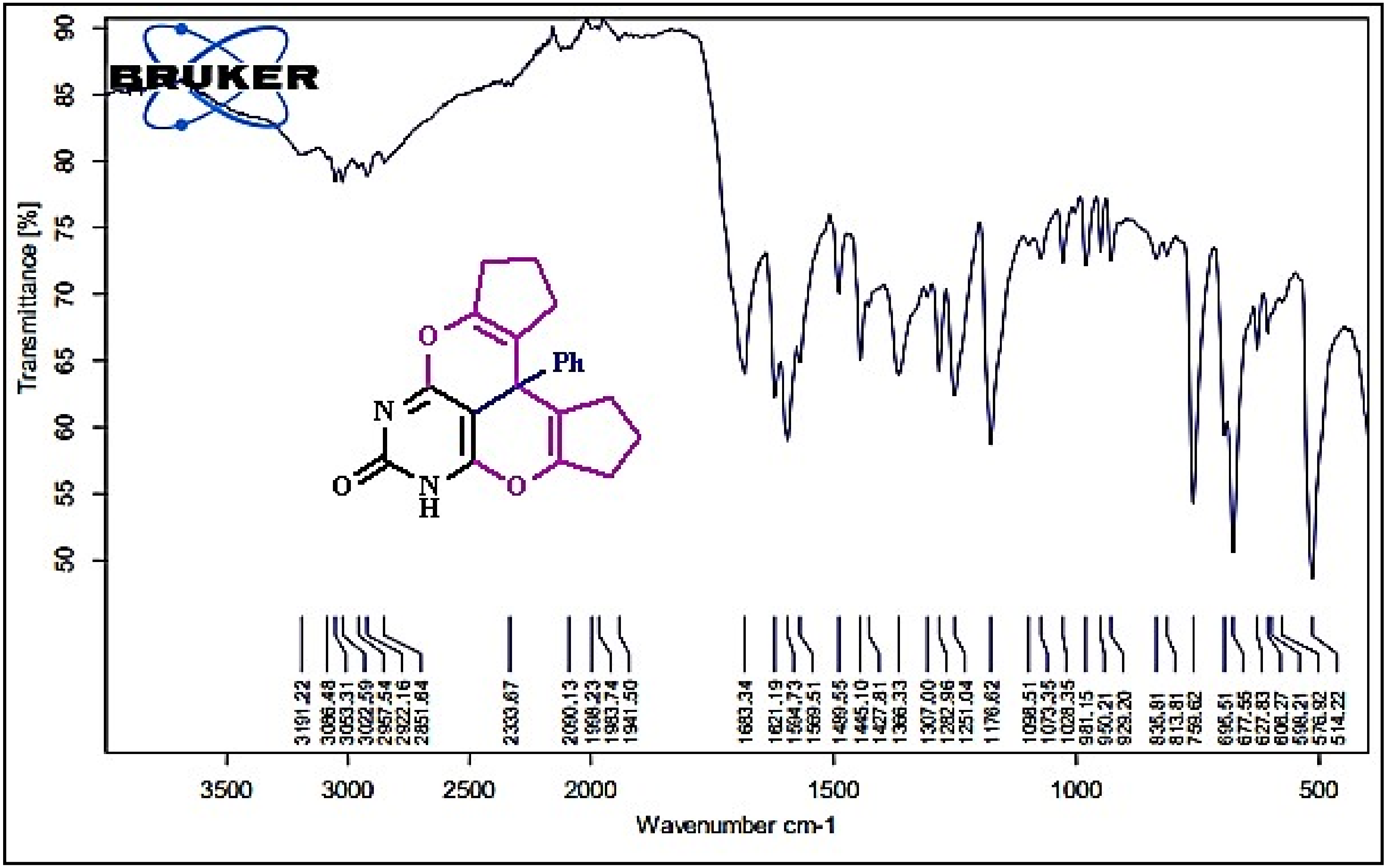


**Fig. 20**: **IR Spectrum of compound 7**

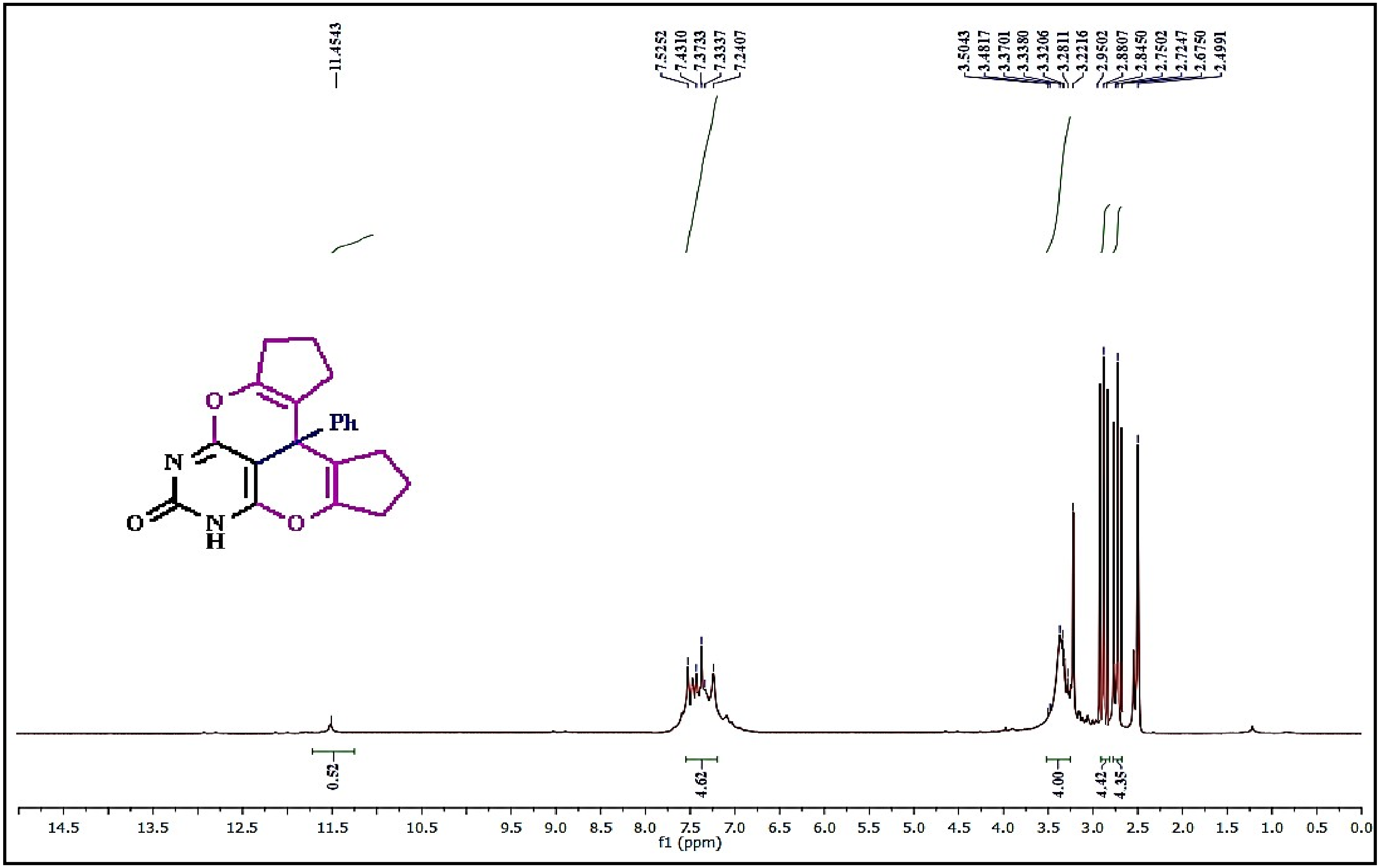


**Fig. 21**: **$^{1}$H-NMR Spectrum of compound 7**

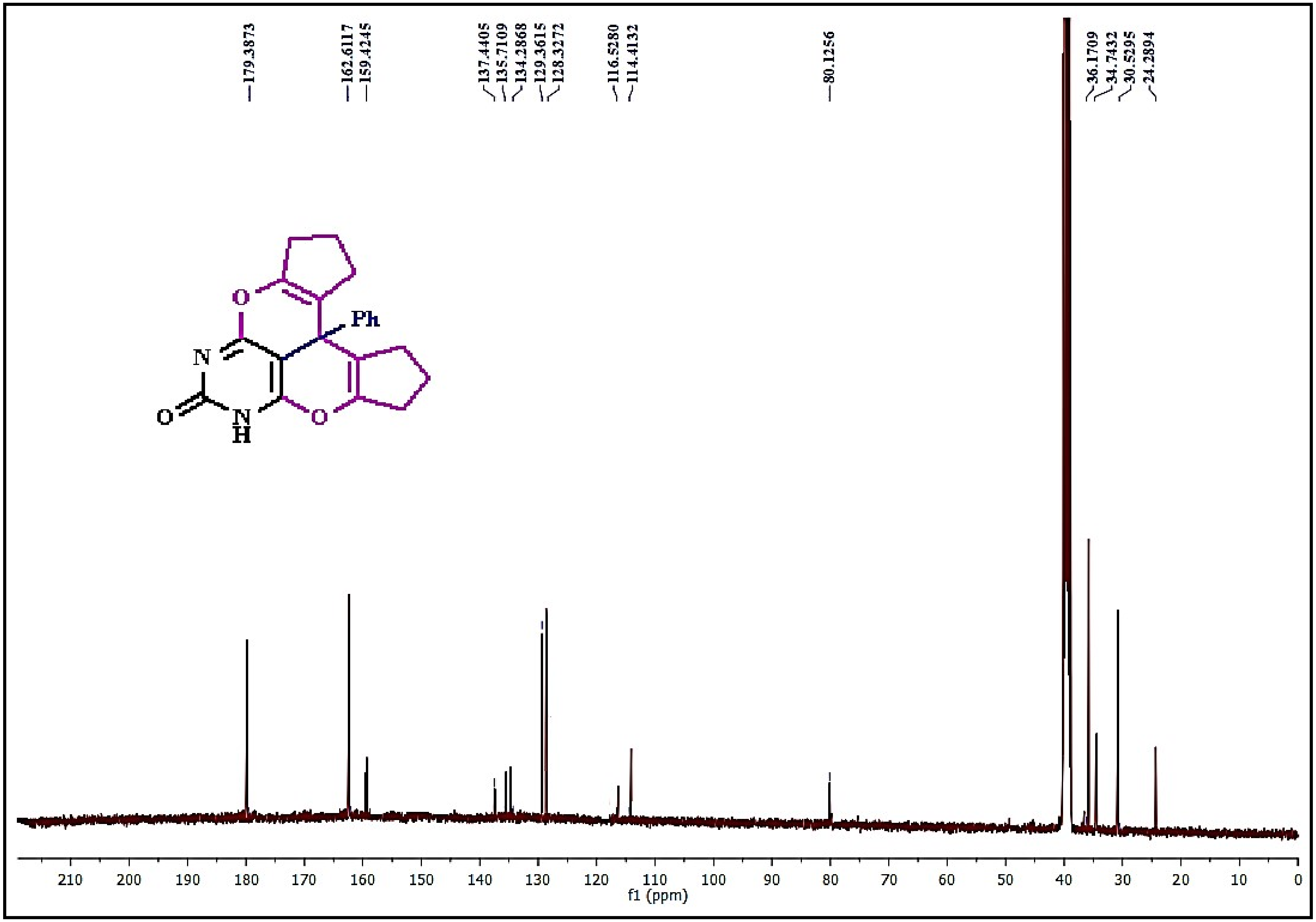


**Fig. 22**: $^{13}$C-NMR Spectrum of compound 7

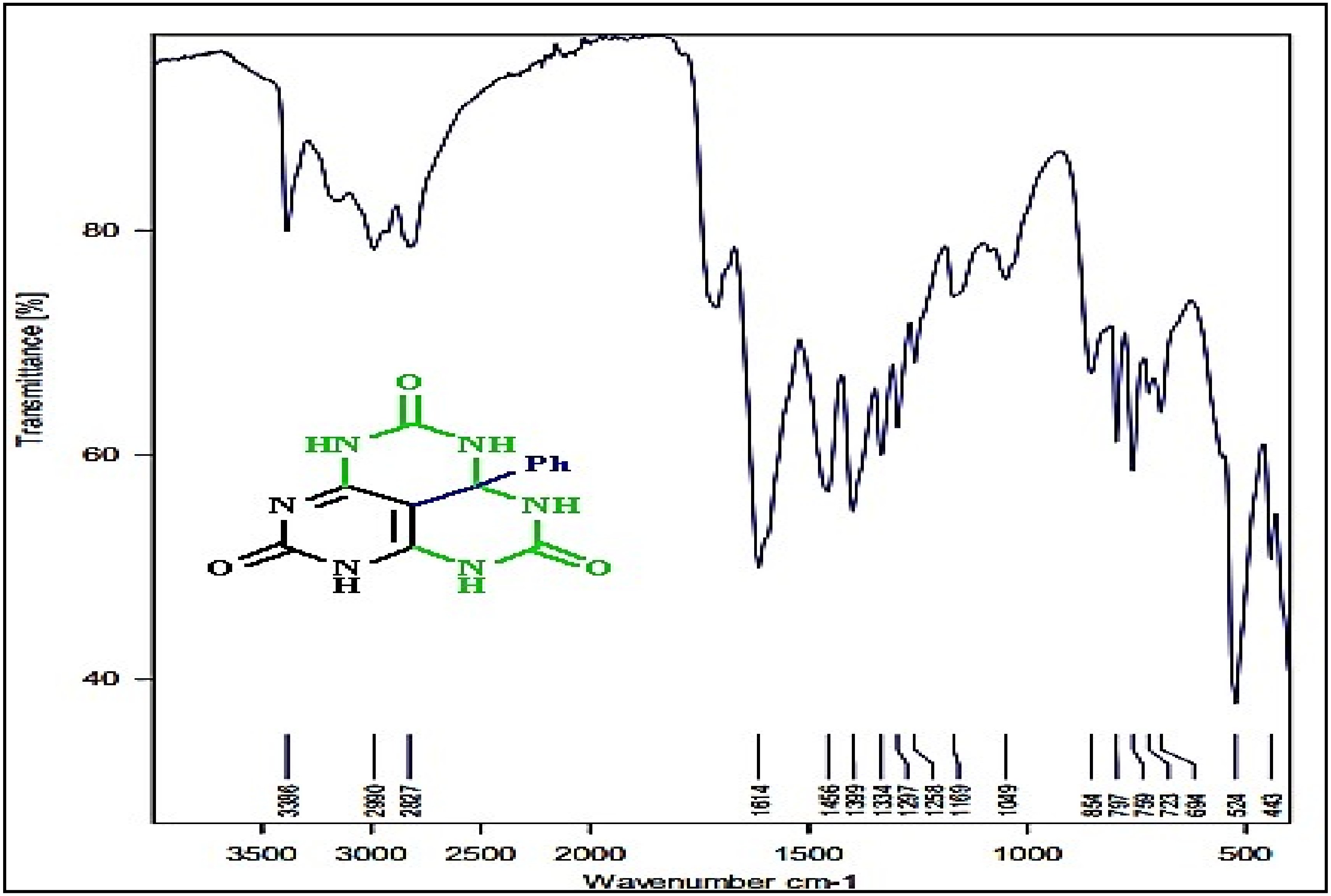


Fig. 23: **IR Spectrum of compound 8**

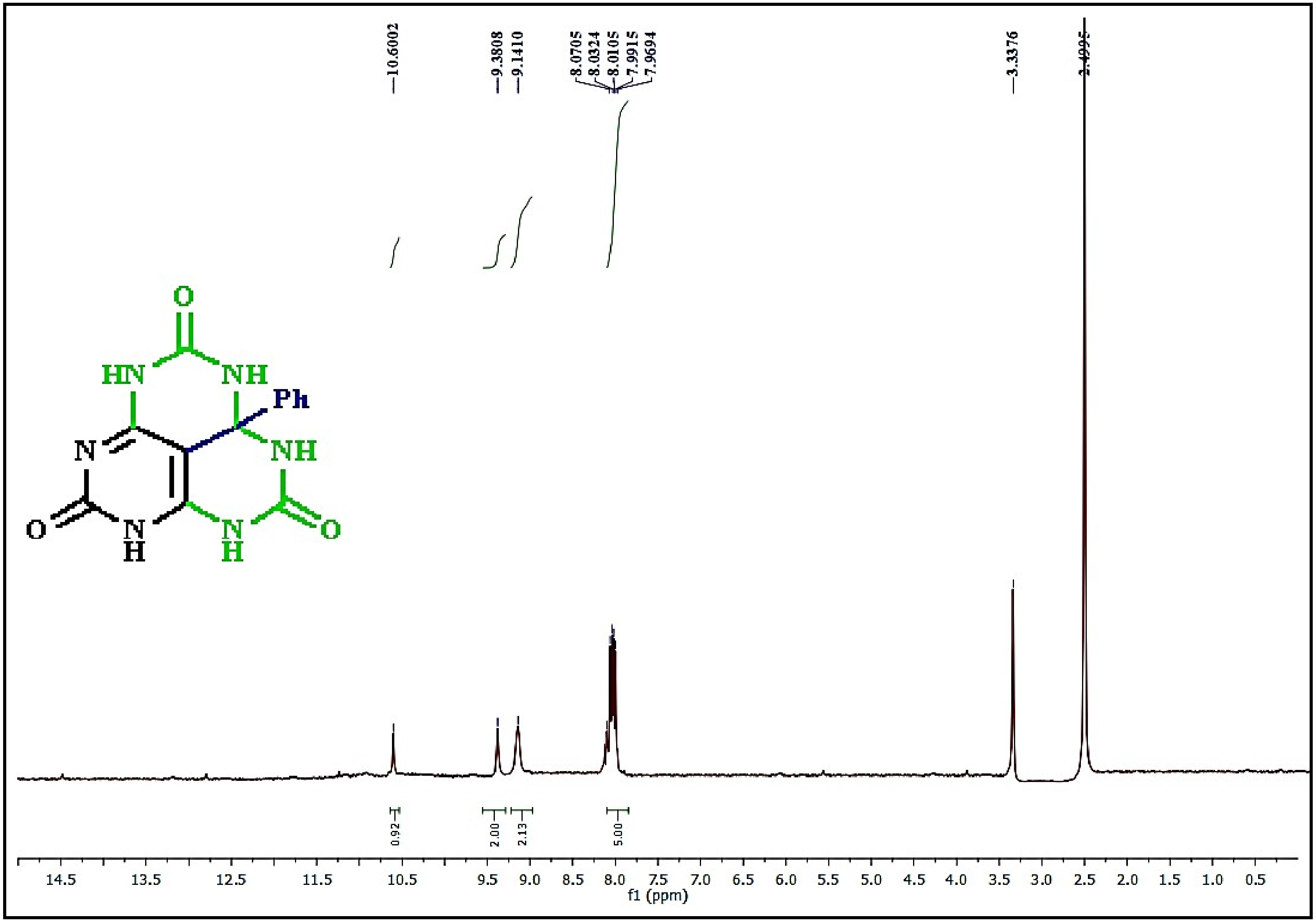


**Fig. 24**: $^{1}$H-NMR Spectrum of compound 8

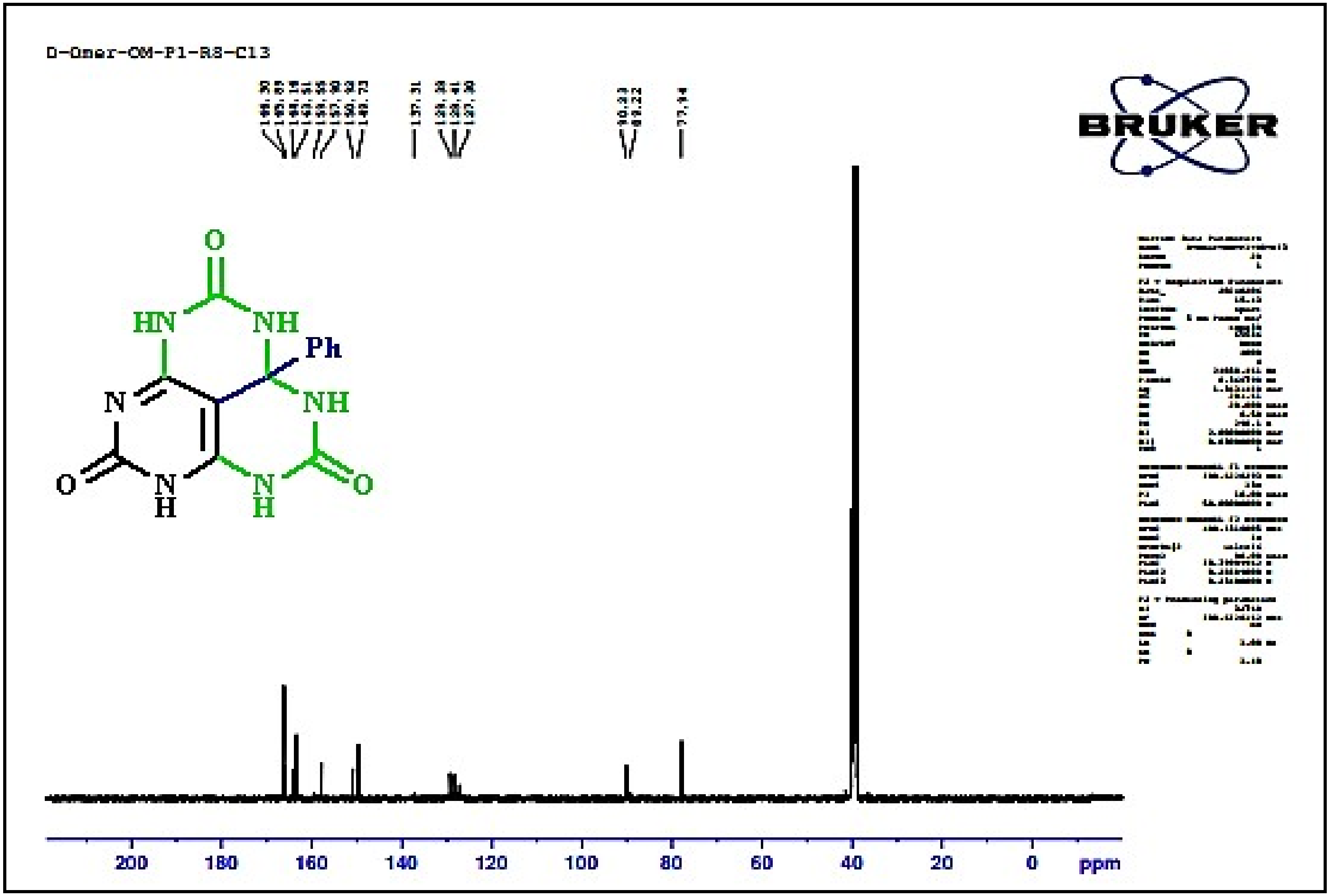


**Fig. 25**: $^{13}$C-NMR Spectrum of compound 8

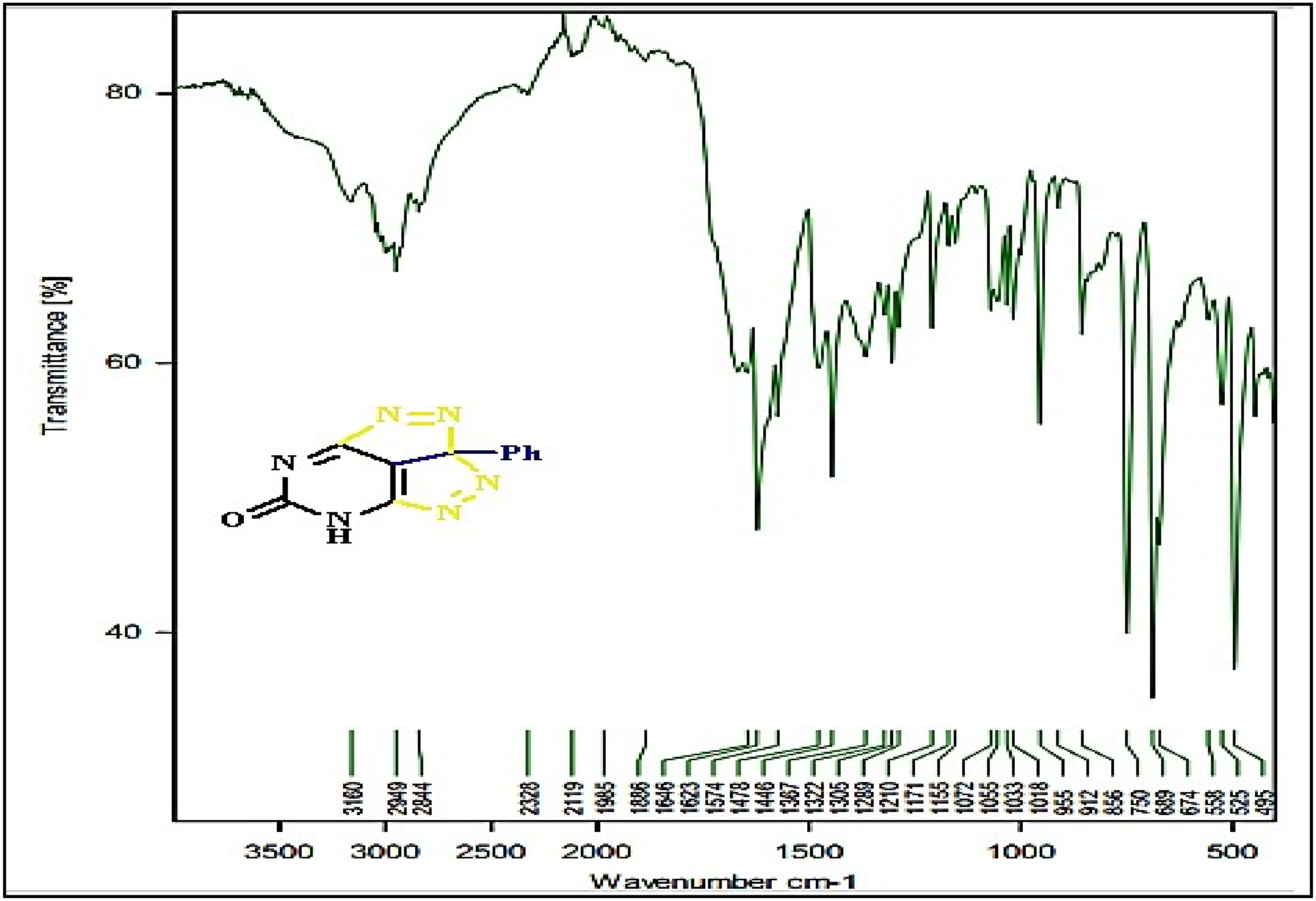


**Fig. 26**: **IR Spectrum of compound 9**

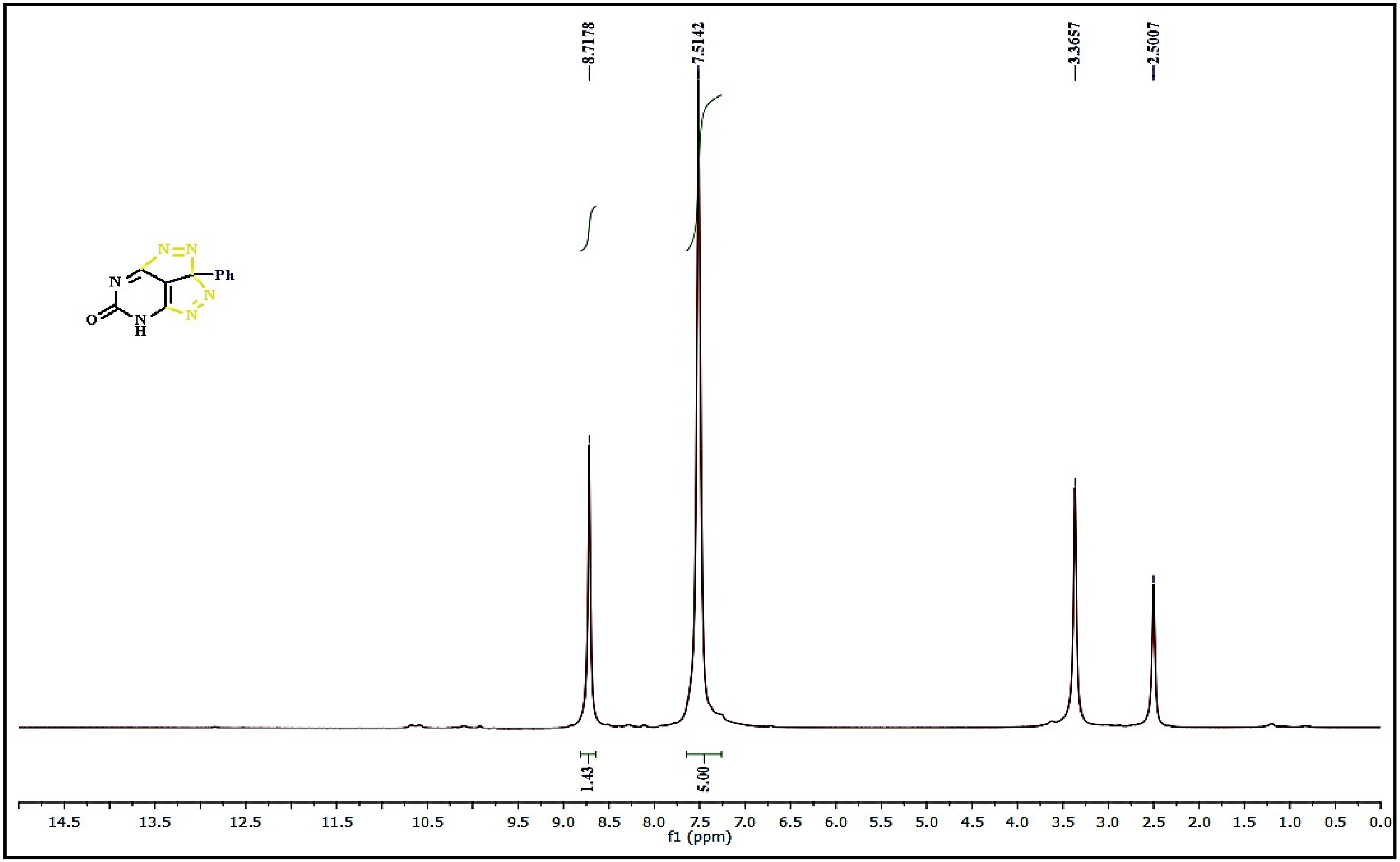


**Fig. 27**: **$^{1}$H-NMR Spectrum of compound 9**

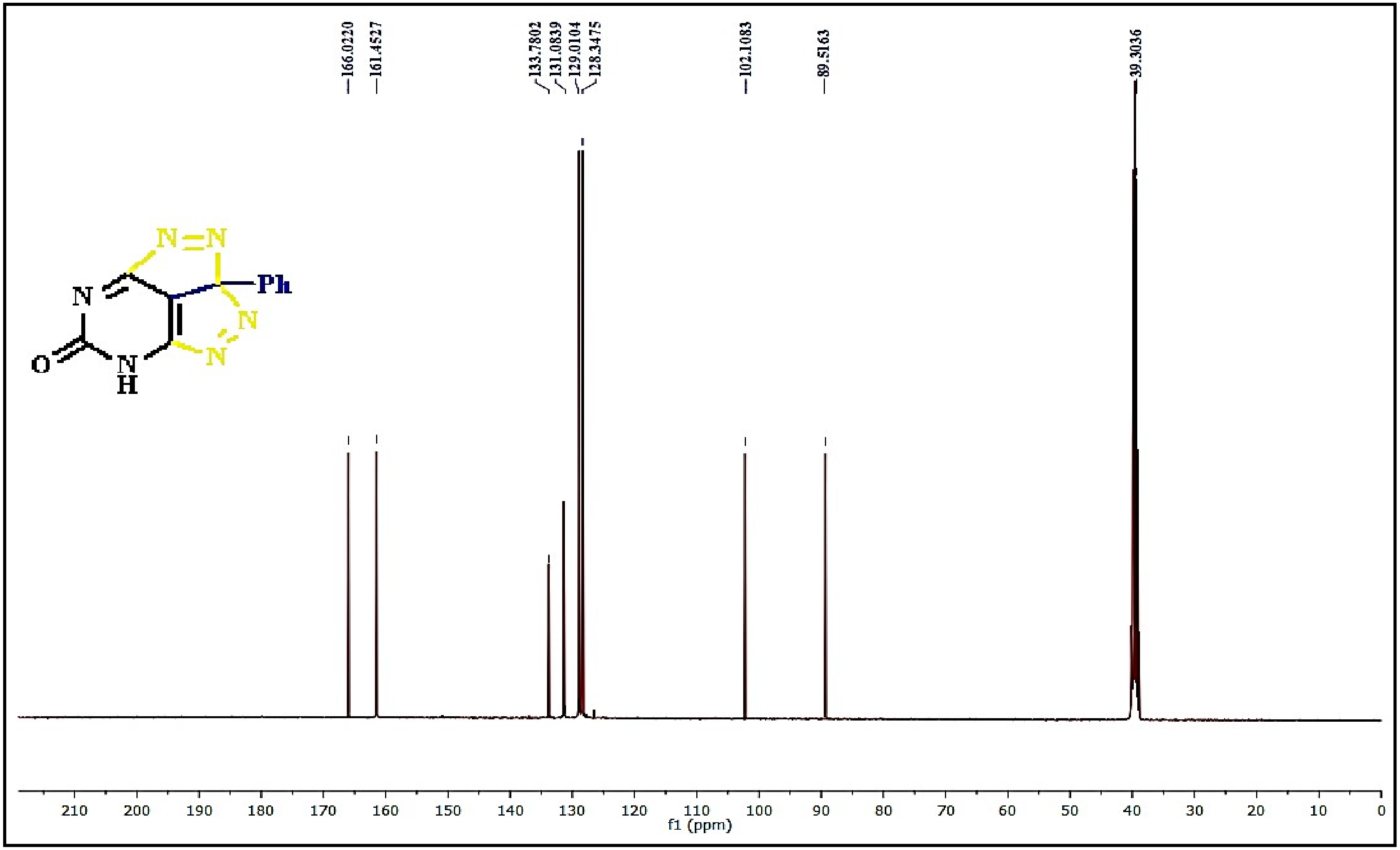


**Fig. 28**: $^{13}$C-NMR Spectrum of compound 9
